# Electrochemically mediated carbon dioxide separation with quinone chemistry in salt-concentrated aqueous media

**DOI:** 10.1038/s41467-020-16150-7

**Published:** 2020-05-08

**Authors:** Yayuan Liu, Hong-Zhou Ye, Kyle M. Diederichsen, Troy Van Voorhis, T. Alan Hatton

**Affiliations:** 1 Department of Chemical Engineering, Massachusetts Institute of Technology, Cambridge, MA 02139 USA; 2 Department of Chemistry, Massachusetts Institute of Technology, Cambridge, MA 02139 USA

**Keywords:** Electrocatalysis, Carbon capture and storage, Electrochemistry

## Abstract

Carbon capture is essential for mitigating carbon dioxide emissions. Compared to conventional chemical scrubbing, electrochemically mediated carbon capture utilizing redox-active sorbents such as quinones is emerging as a more versatile and economical alternative. However, the practicality of such systems is hindered by the requirement of toxic, flammable organic electrolytes or often costly ionic liquids. Herein, we demonstrate that rationally designed aqueous electrolytes with high salt concentration can effectively resolve the incompatibility between aqueous environments and quinone electrochemistry for carbon capture, eliminating the safety, toxicity, and at least partially the cost concerns in previous studies. Salt-concentrated aqueous media also offer distinct advantages including extended electrochemical window, high carbon dioxide activity, significantly reduced evaporative loss and material dissolution, and importantly, greatly suppressed competing reactions including under simulated flue gas. Correspondingly, we achieve continuous carbon capture-release operations with outstanding capacity, stability, efficiency and electrokinetics, advancing electrochemical carbon separation further towards practical applications.

## Abstract (web summary)

Redox-active organic compounds that reversibly bind and release CO_2_ are promising candidates for carbon capture but are limited by the use of flammable, toxic aprotic electrolytes. Here the authors use salt-concentrated aqueous electrolytes in continuous CO_2_ separation with good performance metrics.

## Introduction

Anthropogenic carbon dioxide (CO_2_) emissions present a serious challenge to our society. One of the foremost mitigation strategies involves carbon capture, particularly from large stationary emission sources, followed by sequestration or utilization^1–5^. The incumbent technology for carbon capture is wet chemical scrubbing, which utilizes aqueous amines to chemically absorb CO_2_ and subsequently release it by desorption via a temperature-swing process. However, this process is challenged by the substantial energy demand associated with the regeneration step, sorbent degradation, corrosion, environmental concerns and large footprint^3,6^. To this end, emerging materials such as water-lean solvents and porous solid sorbents have also been garnering widespread attention, with many showing promising capture properties^7–10^. Nevertheless, most of these CO_2_ storing materials are still reliant on high pressure and/or temperature-swing conditions during their operation cycles^10^. Electrochemically mediated separations offer a low-temperature, ambient-pressure alternative for carbon capture, representing a promising yet largely unchartered research area^11–16^. By being electrically driven, these systems can be controlled precisely to reduce energy losses, are modular and thus easy to implement in a variety of locations, and possess great adaptability to the multiscale nature of carbon capture.

In one form of electrochemically mediated carbon capture, redox-active organic compounds serve as CO_2_ carriers. Electrochemical reduction of these compounds generates nucleophiles with a high binding affinity towards electrophilic CO_2_ to afford stable sorbent-CO_2_ adducts; subsequently, the adducts can be oxidized to liberate pure CO_2_ while regenerating the sorbents^13^. To date, several classes of redox-active compounds have been explored, with quinoid species being the most representative example^16–20^. Quinones are a class of cyclic compounds containing two carbonyl groups in an unsaturated six-membered ring structure, which have been studied extensively for their key roles in biological electron transport as well as for promising applications in catalysis and energy storage^21^. They are versatile molecules with highly tailorable electrochemical properties through molecular engineering and can be synthesized from inexpensive commodity chemicals, which is favorable for large-scale applications^22^. Moreover, quinones as CO_2_ sorbents can have an appealingly high theoretical capacity of two CO_2_ equivalents per molecule, compared to conventional wet scrubbing which requires two equivalents of amine to capture one equivalent of CO_2_. Mizen and Wrighton^23^ first reported that electrogenerated 9,10-phenanthrenequinone dianion can chemically react with CO_2_ in homogeneous solutions to yield bis(carbonate), and further studies expanded the quinone chemistries for reversible CO_2_ capture-release^16–18,24,25^. Despite this progress, electrochemically mediated CO_2_ separations still need to be operated using mainly flammable, toxic aprotic organic electrolytes, posing a great barrier toward practical implementation^13^. Recently, the relatively benign ionic liquids have also been explored as electrolytes for electrochemically mediated CO_2_ separations^16,18^. While it may not be a major issue for batch-based separation processes with lean electrolytes, the often-expensive ionic liquids can raise serious cost concerns in continuous-flow absorption systems where they are needed in excess for the dissolution of redox-active CO_2_ carriers.

An aqueous electrolyte is the ideal medium for large-scale electrochemical systems, offering distinct advantages in cost, safety, environmental benignity, and ease of device construction. Carbon capture via redox-active sorbents in aqueous electrolytes is, however, confronted with serious challenges. One critical limitation originates from the narrow electrochemical stability window of water, which is insufficient to support the reductive addition of CO_2_ to quinone molecules without incurring parasitic reactions (hydrogen evolution)^23,26^. Compared to aprotic solvents, aqueous media are complicated by the speciation of CO_2_ upon dissolution, which can undermine the activity of dissolved molecular CO_2_ given inappropriate solution pH^15^. Importantly, various competing reactions with reduced quinone molecules are expected in aqueous solutions, particularly protonation and ion association, hindering severely the kinetics and efficiency of carbon capture^13,27–29^. Finally, rapid evaporative loss is a long-standing concern when interfacing aqueous electrolytes with continuous gas flow, which can seriously compromise the service life of the electrochemical systems^30^.

Herein, by drawing inspiration from the recent breakthrough in salt-concentrated electrolytes for battery applications, we develop an aqueous electrolyte formulation that is fully compatible with quinone-mediated electrochemical carbon capture^26,30–32^. Salt-concentrated aqueous electrolytes, also known as “water-in-salt” electrolytes, are defined as those in which dissolved salt outnumbers water by both mass and volume^26^. In these solutions, the number of water molecules is insufficient to fully solvate the cations so that no free-state water exists, which can effectively suppress the activity of water molecules to extend the electrochemical stability window. By selecting appropriate salt species, desirable solution pH can be realized so that the total dissolved inorganic carbon is composed primarily of molecular CO_2_. It is worthwhile highlighting that, compared to their dilute counterpart, reduced quinone dianions are thermodynamically more reactive towards CO_2_ and less prone to competing reactions in salt-concentrated electrolytes, due to the modified dielectric environment. This results in enhanced carbon capture kinetics and capacity, as verified by both experiments and theoretical calculations. Additional technical superiorities, including high water retention under continuous gas sparging, and diminished dissolution of electrode-immobilized quinone molecules compared to organic electrolytes, are also observed when using our salt-concentrated electrolyte system. These multifold advantages enable the demonstration of quinone-meditated CO_2_ capture-release with excellent capacity, kinetics, reversibility and stability in aqueous media, advancing electrochemical carbon separation further towards practical applications.

## Results

### Electrochemistry of quinone-mediated CO_2_ capture

**Fig. 1:**
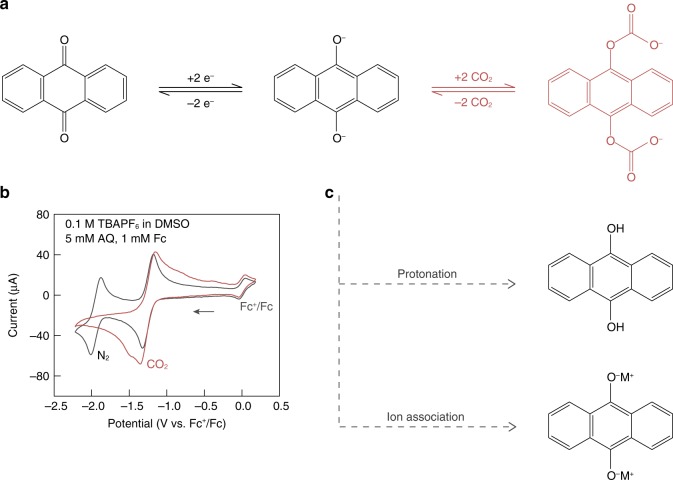
Electrochemistry of quinone-mediated CO_2_ capture. **a** The overall reductive addition reaction between CO_2_ and AQ. **b** CV of AQ in an aprotic organic solvent with and without CO_2_. The CV scans were carried out in 5 mM AQ in dimethyl sulfoxide (DMSO) with 0.1 M tetrabutylammonium hexafluorophosphate (TBAPF_6_) supporting salt. Glassy carbon was used as the working electrode, Pt wire as the counter electrode, Ag wire as a pseudo-reference electrode, and 1 mM ferrocene (Fc) as an internal reference. The scan rate was 50 mV s^−1^. **c** Major competing reactions when carrying out quinone-mediated CO_2_ capture in aqueous media.

Figure 1a shows the overall reaction of quinone-mediated CO_2_ capture and release, using anthraquinone (AQ) as an example. In aprotic media absent of electrophiles, quinones typically undergo two successive one-electron reductions (Fig. 1b, N_2_, and further illustrated in Supplementary Fig. 1), corresponding to the reversible formation of semiquinone radical anion (AQ^•–^) and quinone dianion (AQ^2–^). Interestingly, only a single reduction wave can be observed in the presence of electrophilic CO_2_ and the current nearly doubles with respect to that under N_2_ at the first reduction potential (Fig. 1b, CO_2_), indicating the interaction between reduced quinones and CO_2_. The complexation of CO_2_ with quinone is believed to proceed via an ECEC mechanism (E, electron transfer; C, chemical reaction)^33^. Namely, the reduction of AQ affords AQ^•–^, which can react chemically with CO_2_ through nucleophilic addition; the resulting carbonate renders the quinone aromatic ring relatively neutral such that the second electron transfer can occur at nearly the same potential, generating one additional nucleophilic oxygen for CO_2_ attachment (Supplementary Fig. 2).

Similar electrochemical behavior is also commonly observed in protic electrolytes, where reduced quinones can react with protons to form hydroxyl groups via a two-electron reduction process^22^. Correspondingly, one immediate hurdle when attempting to carry out quinone-mediated carbon capture in aqueous solutions is the rapid protonation of the reduced quinone molecules (Fig. 1c). For example, the rate constant for AQ^2−^ protonation was determined to be twice that of the reaction with CO_2_, such that full utilization of the carbon capture capacity is hardly achievable in the presence of proton donors^27^. Moreover, since both the tendency for quinone protonation and the activity of dissolved molecular CO_2_ increase with increasing solution acidity, an obvious dilemma is presented for aqueous electrolyte formulation. In addition to protonation through the formation of covalent bonds, cations in the supporting electrolyte can also stabilize quinone anions via Coulombic ion pairing (Fig. 1c), which may further reduce the driving force for CO_2_ complexation^29^.

### Aqueous electrolyte for quinone-mediated CO_2_ capture

**Fig. 2:**
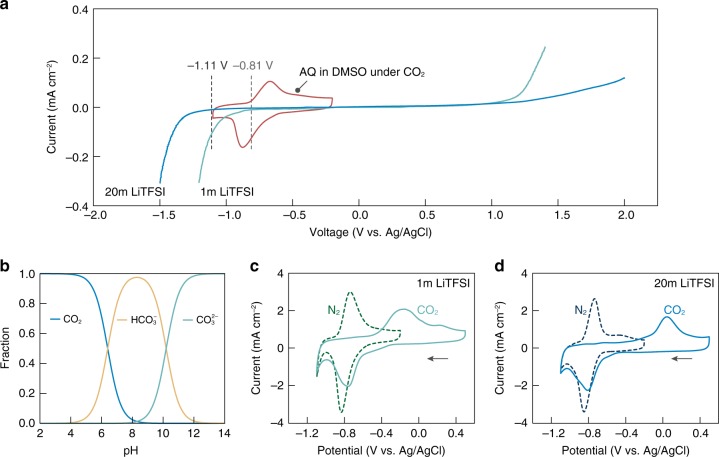
Rational electrolyte formulation for quinone-mediated CO_2_ capture in aqueous media. **a** Linear sweep voltammetry of 1 and 20m LiTFSI aqueous electrolytes at a rate of 1 mV s^−1^, compared with the CV of AQ under CO_2_. Glassy carbon was used as the working electrode. Cathodic and anodic scans were performed separately and joined together afterwards. **b** The Bjerrum plot of the carbonic acid equilibrium as a function of the solution pH for an ideal solution at 25 °C. **c, d** CV of PAQ-CNT in 1m LiTFSI (**c**), and 20m LiTFSI (**d**), under N_2_ (dashed line) and CO_2_ (solid line) at a rate 50 mV s^−1^. PAQ was cast on glassy carbon at a loading of 0.042 mg cm^−2^. For **a**, **c**, **d**, the electrochemical measurements were conducted with Pt wire as the counter electrode and Ag/AgCl as the reference electrode. Source data are provided as a Source Data file.

The prerequisite for quinone-mediated CO_2_ capture-release in aqueous media is a wide electrochemical stability window of the electrolytes. Since only reduced quinones with sufficient electron density can undergo nucleophilic addition with molecular CO_2_, the formation of quinone-CO_2_ adducts generally occurs at potentials more negative than the reductive decomposition (hydrogen evolution reaction) potential of water^33^. Figure 2a compares the cyclic voltammogram (CV) of AQ under CO_2_ with the stability window of 1m LiTFSI aqueous solution (where m is molality and LiTFSI represents lithium bis(trifluoromethanesulfonyl)imide). The reduction peak of AQ occurred at ~0.88 V vs. Ag/AgCl, whereas the electrolyte started to decompose as early as –0.81 V vs. Ag/AgCl (defined as the potential where the current density reaches 0.01 mA cm^–2^).

Salt-concentrated electrolytes, recently gaining momentum in battery research, are proposed to offer an extended electrochemical stability window by eliminating the presence of free-state water through cation solvation^26,30–32^. For example, when the concentration of LiTFSI was increased to an ultrahigh level of 20m, the cathodic decomposition potential of the electrolyte was shifted to –1.11 V vs. Ag/AgCl, which is sufficient to envelop the electrochemical capture and release of CO_2_ by quinones.

To ensure high solubility, salt species with stable and charge-delocalized anions, such as acetate, TFSI, trifluoromethanesulfonate, and bis(fluorosulfonyl)imide, are preferred when formulating water-in-salt electrolytes^31^. Nevertheless, not all of these salts are compatible with electrochemically mediated carbon capture. Gaseous CO_2_ has many possible transformations upon dissolution in water, yet only dissolved molecular CO_2_(aq) is active towards complexation with reduced quinones (Fig. 2b). Therefore, electrolytes for electrochemically mediated carbon capture should have a pH value of less than 6 to ensure the dominance of CO_2_(aq)^15^. The alkaline nature of acetate anion, being the conjugate base of a weak acid, precludes its use. For this proof of concept study, LiTFSI was selected as it is relatively well studied and satisfies this criterion, having a pH near 4 at both 1m and 20m. The water-in-salt definition applies at concentrations above 5m, and at 20m, the ratio between Li^+^ and H_2_O is only ~1:2.78.

To verify the feasibility of quinone-mediated CO_2_ capture in salt-concentrated aqueous media, CV scans were conducted using poly(1,4-anthraquinone)-carbon nanotube (PAQ-CNT) composite under both N_2_ and CO_2_ atmospheres. PAQ was selected for the reduced solubility compared to its molecular counterpart. The immobilization of PAQ on CNT through π−π interaction provides stable anchoring as well as fast electrical path (theoretical capacity = 260 mAh g^−1^, or 9.7 mmol CO_2_ g^−1^; structure shown in Supplementary Fig. 3)^34^. This material has recently been successfully employed by our group to demonstrate electro-swing-based CO_2_ separation in a parallel passage contactor configuration using an ionic liquid as the electrolyte^16^. Note that PAQ shows only a single, broad redox couple under N_2_ instead of two well-defined redox waves (as in the case of solution-phase molecular quinones), which can be ascribed to the slower electron exchange along the polymeric backbone and the hydrogen bonding effect of the aqueous media^28,35^. A substantial difference in electrochemical behavior can be seen in the presence of CO_2_ using LiTFSI-based electrolytes (Fig. 2c, d and at additional concentrations in Supplementary Fig. 4). The pronounced positive shift in the anodic peak is indicative of quinone bis(carbonate) formation, which requires extra driving force to be oxidized compared to quinone dianions. The successful formation of PAQ-CO_2_ adduct is also confirmed via X-ray photoelectron spectroscopy (XPS), where the C1s spectrum of PAQ reduced in 20m LiTFSI under CO_2_ showed a close resemblance to that of PAQ-CO_2_ adduct obtained using a conventional organic electrolyte (Supplementary Fig. 5). Interestingly, the oxidation potential of the PAQ-CO_2_ adduct increases with increasing LiTFSI concentration. This is attributed to the stronger stabilization effect of Li^+^ on the adduct at higher solution molalities as confirmed by the density functional theory (DFT) calculations (Supplementary Fig. 6 and Supplementary Note 1).

### Minimized competing reactions in salt-concentrated electrolyte

**Fig. 3:**
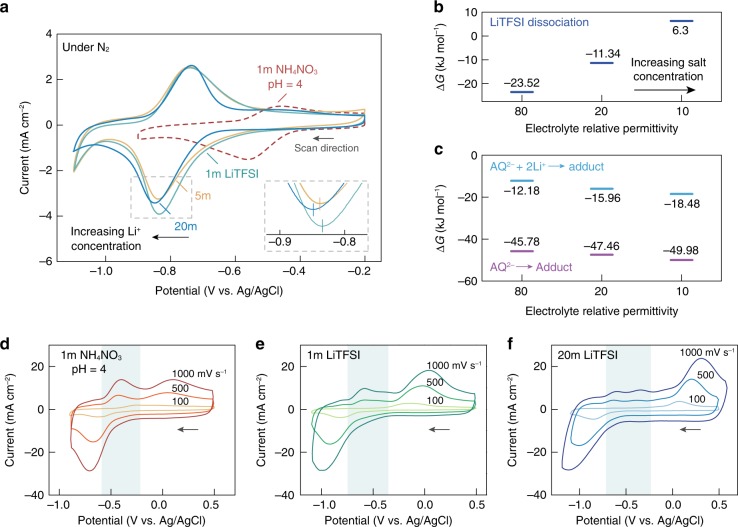
Enhanced reactivity of CO_2_ with quinones in salt-concentrated media. **a** CV of PAQ-CNT in 1m NH_4_NO_3_, 1, 5 and 20m LiTFSI under N_2_. The scans were conducted at a rate 50 mV s^−1^. **b** The free energy change for LiTFSI dissociation at different electrolyte permittivities. Relative permittivities of 80 and 10 correspond to 1 and 20m LiTFSI, respectively. The process becomes nonspontaneous at high LiTFSI concentrations. **c** The free energy change for quinone-CO_2_ adduct formation at different electrolyte permittivities, starting from both AQ^2–^ and AQ^2–^ complexed with Li^+^. The adduct formation becomes thermodynamically more favorable at higher LiTFSI concentrations. **d**−**f** CV of PAQ-CNT at different scan rates in 1m NH_4_NO_3_ (**d**), 1m LiTFSI (**e**), and 20m LiTFSI (**f**) under CO_2_. The shaded area corresponds to the oxidation of quinone dianions that are unreacted with CO_2_. For all the CV measurements, PAQ was cast on glassy carbon at a loading of 0.042 mg cm^−2^, Pt wire was used as the counter electrode, and Ag/AgCl was used as the reference electrode. Source data are provided as a Source Data file.

To understand the effect of possible competing reactions on the reactivity of PAQ with CO_2_, Fig. 3a shows the CV scans of PAQ under N_2_ in 1m NH_4_NO_3_ (pH adjusted to 4 with HNO_3_) and LiTFSI electrolytes with different molalities (pH ~ 4). It was previously established that protonation and cation association can stabilize reduced quinone species, inducing anodic shifts in the quinone reduction potential^28^. The reduction potential of PAQ in 1m NH_4_NO_3_ is significantly more positive than those in LiTFSI-based electrolytes. As NH_4_^+^ is generally regarded as a bulky cation with low tendency for anion association, the large anodic shift in the reduction potential in 1m NH_4_NO_3_ can only be ascribed to the protonation of reduced PAQ^36^. The protonation of reduced PAQ in 1m NH_4_NO_3_ electrolyte severely undermines its potential carbon capture ability, which can be corroborated by ill-defined adduct oxidation peaks in CV scans under CO_2_ (Supplementary Fig. 7).

Protonation is greatly suppressed in LiTFSI-based electrolytes where Li^+^ significantly outnumbers H^+^, rendering ion association the major competing reaction. Notably, though, both the onset and the peak potential of PAQ reduction shift towards more negative values with increasing LiTFSI concentrations (Fig. 3a), indicating weakened ion association. This is likely attributed to the dominance of associated salt species (contact ion pairs, CIP, and aggregates) in concentrated solutions as a result of incomplete cation solvation by water molecules^26,31^. To confirm this mechanistic interpretation, the free energy change (Δ*G*) for LiTFSI dissociation was calculated by DFT, where the increase in salt concentration can be modeled as a decrease in the dielectric constant of the electrolyte (Fig. 3b, Supplementary Fig. 8)^37^. The DFT results confirmed that the dissociation of LiTFSI becomes nonspontaneous (Δ*G* > 0) above a certain concentration threshold, such that the kinetics of Li^+^-involved processes are necessarily slowed down in a salt-concentrated environment. More importantly, the thermodynamic driving force for quinone-CO_2_ adduct formation, from both AQ^2−^ and AQ^2−^ complexed with Li^+^, also increases with increasing electrolyte concentrations based on the DFT calculations (Fig. 3c).

This conclusion of enhanced reactivity of CO_2_ with quinones in salt-concentrated media can be seen experimentally by CV measurements under different scan rates (Fig. 3d−f). Interestingly, besides the oxidation of quinone-CO_2_ adduct, an additional oxidation peak at less positive potential can be seen during fast scans (shaded area in Fig. 3d−f), corresponding to the oxidation of quinone dianions that did not chemically react with CO_2_. Such a phenomenon is caused by the much more rapid electrochemical generation of reduced quinone species compared to the relatively slow CO_2_ transport and chemical reaction with reduced quinones, which inevitably becomes more pronounced with increasing scan rates. Quantitatively, while 100% of the electrochemically generated quinone dianions reacted with CO_2_ at a scan rate of 100 mV s^–1^ in LiTFSI-based electrolytes, the value was only 77.5% when using 1m NH_4_NO_3_ likely due to the rapid protonation; and compared to 1m LiTFSI, a higher percentage of adduct formation was observed in 20m LiTFSI under all scan rates, thanks to the suppressed Li^+^ association (Supplementary Fig. 9).

Therefore, it is clear from the above discussion that the reductive addition of CO_2_ to quinones is much less susceptible to competing reactions when carried out in salt-concentrated aqueous media, making them particularly attractive for quinone-mediated carbon capture.

### Physicochemical advantages of salt-concentrated electrolyte

In addition to being fully compatible with quinone electrochemistry and minimizing competing reactions, concentrated LiTFSI aqueous electrolytes also possess favorable physicochemical properties for the practical applications of electrochemically mediated carbon capture.

**Fig. 4:**
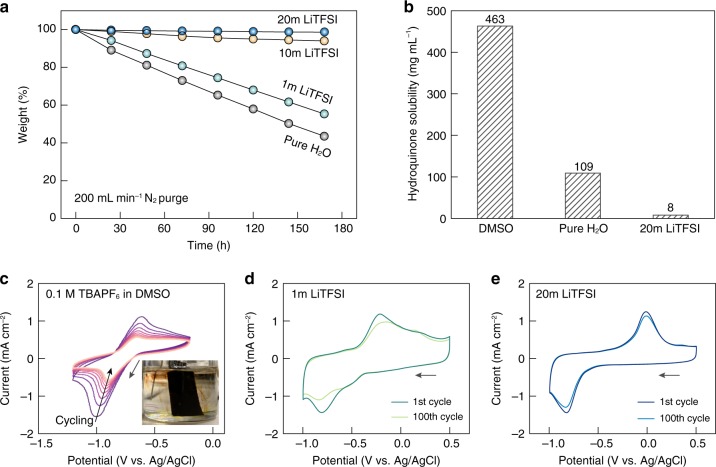
Physicochemical advantages of salt-concentrated electrolytes for quinone-mediated CO_2_ capture. **a** Weight retention curves of different electrolytes under constant N_2_ sparging at a high flow rate of 200 mL min^−1^. **b** Hydroquinone solubility in a representative organic solvent (DMSO), pure water and 20m LiTFSI aqueous electrolyte. **c** Multicycle CV scans of PAQ-CNT in 0.1 M TBAPF_6_ DMSO electrolyte at a rate of 100 mV s^−1^. Inset shows the severe dissolution of PAQ (immobilized on carbon felt) into the electrolyte during electrochemical reduction in a DMSO-based electrolyte. **d**, **e** The 1st and 100th CV scans of PAQ-CNT under CO_2_ in 1m LiTFSI (**d**) and 20m LiTFSI (**e**) at a rate of 50 mV s^−1^. For all the CV measurements, PAQ was cast on glassy carbon, Pt wire was used as the counter electrode, and Ag/AgCl was used as the reference electrode. Source data are provided as a Source Data file.

Firstly, salt-concentrated aqueous media demonstrate significantly enhanced resistance to evaporative loss compared to their dilute counterparts. Figure 4a plots the weight evolution of LiTFSI-based electrolytes under continuous, high-flow-rate gas sparging, where a stark contrast in water retention capability exists between the dilute (pure H_2_O, 1m LiTFSI) and the concentrated (10 and 20m LiTFSI) electrolytes. While pure water suffered from >56% weight loss (with respect to the initial electrolyte weight) after 168 h of gas purging, the value was merely 1.3% for 20m LiTFSI. This unique feature effectively dispels the traditional concern of electrolyte dry-out when interfacing aqueous solutions with gas flows, rendering the potential quinone-mediated carbon capture system with flexibility in form factor and durability.

Secondly, salt-concentrated aqueous media containing no free water inhibit the dissolution of electroactive compounds immobilized on heterogeneous substrates, which is desirable for capacity retention during electrochemical cycling of many systems. Quinones, especially in their reduced forms, generally show high dissolution tendency in polar electrolytes^24,35,38^. Correspondingly, hydroquinone was selected as a probe molecule, and its solubility in various electrolytes was determined via UV−Vis spectrometry (Fig. 4b, Supplementary Figs. 10 and 11). A high hydroquinone solubility of 463 mg mL^−1^ was observed in dimethyl sulfoxide (DMSO) which is a representative organic solvent, resulting in visible PAQ dissolution and rapid capacity decay during electrochemical cycling (Fig. 4c and Supplementary Fig. 12). The solubility was decreased by fourfold when the organic solvent was replaced with water, yet the value remained significant. Therefore, ~35% capacity loss of PAQ-CNT (decrease in cathodic peak intensity) was seen after 100 CV cycles in 1m LiTFSI (Fig. 4d). Noticeably, compared to that in pure water, the solubility of hydroquinone was reduced even further by ~14 times in 20m LiTFSI. The greatly suppressed PAQ solubilization in salt-concentrated electrolytes, together with minimized side reactions, enabled impressive PAQ cycling stability (~90% capacity retention after 100 CV cycles, Fig. 4e).

### Electrochemical cycling with high capacity and stability

To further evaluate the electrochemical performance of quinone-mediated CO_2_ capture-release in salt-concentrated aqueous media, PAQ-CNT was immobilized on porous carbon felt substrates to increase both the active material mass loading and the electrode−electrolyte contact area. Fourier-transform infrared spectroscopy (FTIR) was employed to quantify the CO_2_ uptake capacity (Supplementary Fig. 13). Specifically, the PAQ-CNT electrode was electrochemically reduced to capture CO_2_ and was then placed in a sealed vial for thermal regeneration. After release, the headspace was sampled using an FTIR gas cell to determine the CO_2_ concentration. The thermal release capacity (CO_2_ per gram of PAQ) was ~4.7 mmol g^−1^ at 80 °C and increased to ~6.2 mmol g^−1^ at 120 °C. Nevertheless, as observed in previous studies^24,25^, thermal regeneration is less effective than the electrochemical route such that only ~70% of the captured CO_2_ was liberated at 120 °C (Supplementary Fig. 14). Considering this thermal release inefficiency, the carbon capture capacity of PAQ is ~8.9 mmol g^−1^, which is close to its theoretical value of 9.7 mmol g^−1^.

**Fig. 5:**
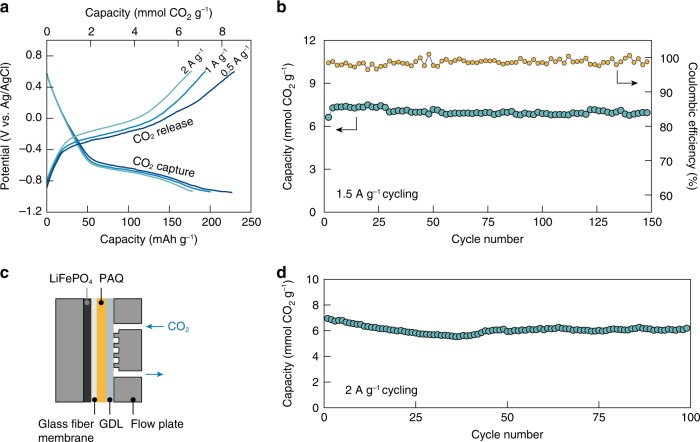
Electrochemical carbon capture and release with high capacity and stability. **a** Electrochemical CO_2_ capture-release voltage profiles at different cycling current densities in 20m LiTFSI. **b** Long-term cycling stability and Coulombic efficiency of PAQ-CNT in 20m LiTFSI at a high current of 1.5 A g^−1^ (capture/release in ~10 min). For (**a**) and (**b**), the mass loading of PAQ on carbon felt was 0.2 mg cm^−2^, LiFePO_4_ on Al foil was used as the counter electrode, Ag/AgCl was used as the reference electrode, and the measurements were carried out in a conventional single-compartment electrochemical cell. **c** Schematic illustrating the configuration of the membrane flow cell for CO_2_ capture-release. PAQ was immobilized on carbon felt at a mass loading of 1 mg cm^−2^, LiFePO_4_ coated on Al foil was used as the counter electrode, and the two electrodes were separated by an electrolyte-imbibed glass fiber membrane. A hydrophobic gas diffusion layer (GDL) was placed on one side of the PAQ electrode and interfaced with continuous CO_2_ flow. **d** Long-term cycling stability of the membrane flow cell in 20m LiTFSI at a cycling current of 2 A g^−1^. For (**b**) and (**d**), PAQ reduction was carried out at constant current with a cut-off voltage of either −0.95 V vs. Ag/AgCl or −1.4 V vs. LiFePO_4_. The oxidation of PAQ-CO_2_ was conducted via the CC/CV mode (constant current followed by constant voltage). The cut-off voltage for CC oxidation was either 0.2 V vs. Ag/AgCl or −0.1 V vs. LiFePO_4_; and CV oxidation was carried out until the current decayed to 10% of the CC oxidation current. Source data are provided as a Source Data file.

Figure 5a shows the electrochemical CO_2_ capture-release voltage profiles at different cycling currents in 20m LiTFSI electrolyte, where the PAQ electrode demonstrated excellent charge transfer kinetics. When cycled at a high current of 0.5 A g^−1^ (capture/release in ~30 min), a carbon capture capacity of ~8.4 mmol g^−1^ can be achieved (>86% of the theoretical value), which is higher than in the previously reported quinone-mediated carbon capture systems. Moreover, the value is on par with, if not higher than, the established amine-based processes (~8 mmol per gram of amine, but ~1−2 mmol per gram of the total scrubbing absorbent)^24,39^. Importantly, the capacity was only affected slightly with further increase in the cycling currents. For example, a working capacity of ~6.6 mmol g^−1^ could still be obtained when the capture/release process was completed in only ~6 min (2 A g^−1^). The quinone-mediated CO_2_ capture-release also demonstrated outstanding cycling stability and reversibility in salt-concentrated aqueous electrolytes (Fig. 5b). Minimal capacity loss can be observed after 150 cycles at a high current of 1.5 A g^−1^ (capture/release in ~10 min), with an average Coulombic efficiency (release capacity/capture capacity) of near unity.

To gain deeper insights into the electrochemical CO_2_ capture-release performance in a practical scenario, a prototypical membrane-based flow cell was constructed as shown schematically in Fig. 5c. LiFePO_4_ was chosen as the counter electrode due to its well-established Li^+^ intercalation−deintercalation electrochemistry in aqueous environments (~0.42 V vs. Ag/AgCl in 20m LiTFSI) with high reversibility and excellent electrokinetics^39,40^. The capacity of the LiFePO_4_ electrode was at least four times in excess of that of the PAQ-CNT electrode to minimize the polarization from the counter electrode. The shortened ion-transport distance between the electrodes and the continuous delivery of gas-phase CO_2_ enabled high-current carbon capture-release with increased active material mass loading^41^. At a current of 2 A g^−1^ and a mass loading of 1 mg cm^−2^, the membrane flow cell demonstrated over 100 cycles of continuous carbon capture and release with good capacity retention (Fig. 5d), highlighting the outstanding capacity, stability, and reversibility of quinone-mediated carbon capture in salt-concentrated aqueous media.

### CO_2_ response during electrochemical quinone cycling

**Fig. 6:**
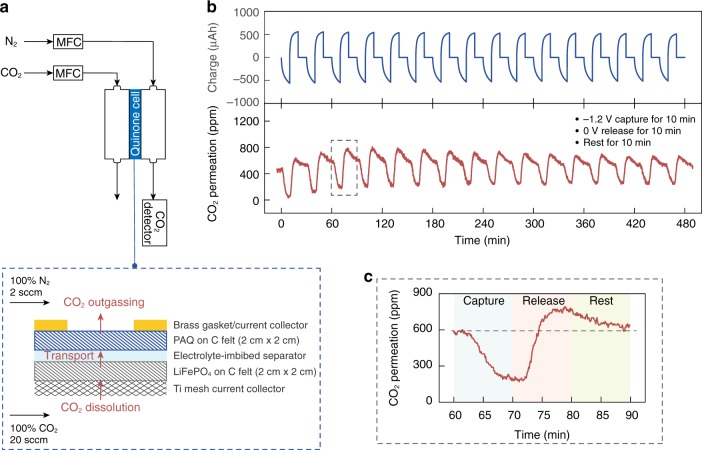
CO_2_ response during electrochemical quinone cycling. **a** Schematic illustrating the setup for the in situ measurement of CO_2_ response during PAQ cycling. MFC stands for mass flow controller. The electrochemical cell consisted of PAQ-CNT (PAQ 1 mg cm^−2^) and LiFePO_4_ (8 mg cm^−2^) electrodes cast on 50 μm-thick carbon felt (2 cm × 2 cm in area), and the two electrodes were separated by a 20m LiTFSI-imbibed polypropylene separator. The electrochemical cell was mounted on a brass gasket with a 1 cm^2^ circular opening to the gas flow chamber, which also served as the current collector for the PAQ-CNT electrode. A titanium mesh with little impedance to gas flow was used as the current collector for LiFePO_4_. Pure CO_2_ flowed on one side of the electrochemical cell and pure N_2_ flowed on the other side to carry permeated CO_2_ to the in-line gas detector. The testing employed a “zero-gap” design where the electrochemical components were all in close contact, such that CO_2_ cross-over can only occur via dissolution-diffusion through the electrolyte. **b** CO_2_ permeation in response to the electrochemical cycling of the PAQ-LiFePO_4_ cell, and the corresponding oxidation/reduction charge profiles. **c** Detailed CO_2_ permeation behavior during one capture-release cycle marked with a dashed line in (**b**). The cycling was conducted following a constant voltage capture-release protocol (capture at −1.2 V for 10 min, release at 0 V for 10 min, rest 10 min). Source data are provided as a Source Data file.

Finally, we constructed a gas device to directly monitor the CO_2_ response in situ during quinone electrochemical cycling. The experimental setup is shown in Fig. 6a. Briefly, the PAQ-LiFePO_4_ electrochemical cell was mounted on a gasket with CO_2_ flowing on one side. Gaseous CO_2_ can dissolve into the electrolyte, transport through the electrochemical cell via diffusion and outgas into the N_2_ sweep stream on the other side. The permeated CO_2_ was then carried towards an in-line gas analyzer to determine the concentration. The detailed configuration of the gas cell is provided as Supplementary Fig. 15.

Figure 6b shows the CO_2_ concentration response of the gas device during constant voltage capture and release (each capture/release step was 10 min, followed by a 10-min rest), where a cyclic fluctuation of the permeated CO_2_ concentration can indeed be clearly observed. During capture, CO_2_ dissolved in the electrolyte was being actively consumed by the PAQ-CNT electrode, resulting in a continuous decrease in CO_2_ permeation; and the subsequent electrochemical oxidation of the PAQ-CO_2_ adduct generated CO_2_ rapidly, leading to a sharp concentration spike until all the captured CO_2_ was released and the permeation restored to its background value (Fig. 6c). Note that the discrepancy between the onset of the electrochemical capture/release and the CO_2_ signal response was due to system dispersion and the headspace volume of the flow chamber. Importantly, the consistent CO_2_ response over repeated electrochemical cycling further confirmed the stability of quinone chemistry for carbon capture in salt-concentrated aqueous media.

### System testing in the presence of oxygen

The potential parasitic reaction (peroxide formation) between reduced quinones and molecular oxygen (O_2_) present in the feed stream is generally regarded as a source of inefficiency in our described CO_2_ separation scheme^13,17^. Therefore, the electrochemical behavior of quinones in salt-concentrated aqueous electrolytes was further evaluated under mixed CO_2_/O_2_ conditions.

It is worth emphasizing that electrochemically mediated carbon capture operates in a mass transfer-limited regime. Therefore, we can take advantage of the substantial solubility difference between O_2_ and CO_2_ to reduce the efficiency loss. For aqueous solutions at 25 °C, CO_2_ has ~26 times higher solubility than O_2_ under the same partial pressure^42^. While application for dilute scenarios such as direct air capture requires further investigation, we believe redox-active sorbents are competitive for carbon capture from stationary sources with relatively high CO_2_ partial pressure, such as carbon capture from power plants and large industrial processes (steel, cement and fertilizer plants, etc.), as well as in nonoxidizing environments (natural gas and biogas upgrading, etc.), which account for ~60% of the total global greenhouse gas emissions^43^. Flue gas from coal-fired power plants typically contains 12−15% CO_2_ and 3−5% O_2_; in other industrial plants, the CO_2_ concentration in exhaust streams can be even higher (e.g. cement production 15−30%, iron and steel 20−44%)^43^. Therefore, the limiting current involving CO_2_ can be two orders of magnitude higher than that involving O_2_ in our targeted carbon capture scenarios.

**Fig. 7:**
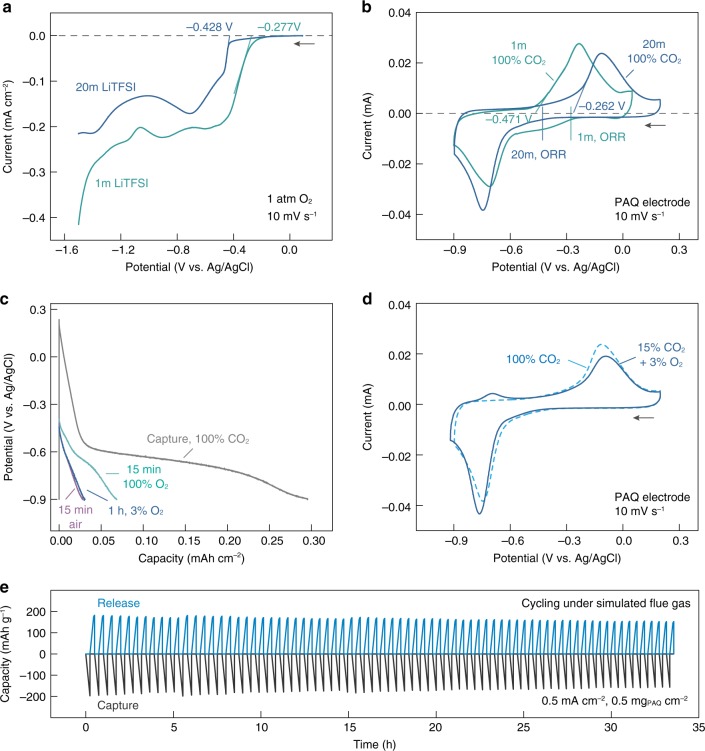
Electrochemical CO_2_ capture-release in the presence of O_2_. **a** Linear sweep voltammograms of 1 and 20m LiTFSI saturated with O_2_ measured with a glassy carbon electrode at a rate of 10 mV s^−1^. **b** Comparison between the oxidation potential of PAQ-CO_2_ (with onset potential noted) and the ORR potential (from **a**) in 1 and 20m LiTFSI. **c** Reduction profiles of PAQ under CO_2_ in 20m LiTFSI and of the same electrode after being rested in 100% O_2_ (15 min), air (15 min) and 3% O_2_ (1 h), respectively. The reductions were carried out at a current density of 0.5 mA cm^−2^ and the PAQ was immobilized on carbon felt at a mass loading of 1.2 mg cm^−2^. **d** CV scans of PAQ under pure CO_2_ and simulated flue gas in 20m LiTFSI. The ratio between the total anodic and cathodic capacities was 87.3% and 80.7% for pure CO_2_ and simulated flue gas, respectively. The additional anodic peak observed under simulated flue gas corresponds to the oxidation of quinone anions that did not react with CO_2_ due to mass transport limitation. For (**b**) and (**d**), the CV scans were conducted at a rate of 10 mV s^−1^ with 1.5 μg PAQ loaded on the glassy carbon electrode. **e** The capture-release capacity of PAQ during continuous cycling under simulated flue gas. The cycling was carried out in the membrane flow cell configuration at a current density of 0.5 mA cm^−2^ and a PAQ mass loading of 0.5 mg cm^−2^ (immobilized on carbon felt). CC capture and CC/CV release protocol was adopted with the cut-off voltage being −1.35 and −0.1 V, respectively. Each capture/release step has a time scale of ~15 min. Source data are provided as a Source Data file.

The potential of the O_2_/O_2_^2−^ redox couple in 1 and 20m LiTFSI was studied using a glassy carbon electrode, which is known to catalyze the two-electron oxygen reduction reaction (ORR, Fig. 7a). The ORR onset potential was relatively negative for both cases, which can be attributed to the limited proton availability. The reaction product is likely to be Li_2_O_2_, which has been observed to be stable in salt-concentrated aqueous media due to significantly reduced H_2_O activity^40^, making it a poor oxidant. Importantly, compared to 1m LiTFSI, the onset potential was 150 mV more negative in 20m LiTFSI together with reduced limiting current, further lowering the driving force for parasitic ORR. No obvious differences in CV can be observed for PAQ in 20m LiTFSI under N_2_ and O_2_ (Supplementary Fig. 16a). The ratio between the anodic and cathodic peak currents (I_pa_/I_pc_) is 0.98 and 0.97 under N_2_ and O_2_ respectively, confirming the relatively low sensitivity of reduced PAQ towards O_2_ in 20m LiTFSI. Redox-active sorbents with higher reduction potentials (e.g. 1,4-naphthoquinone, NQ, Supplementary Fig. 16b) can also be chosen to minimize side reactions.

Importantly, the quinone-CO_2_ adducts are much more stable against oxidation compared to the corresponding quinone dianions due to their increased oxidation potential and, as described previously, the oxidation potential also increases with increasing LiTFSI concentration (Fig. 2c, d and Supplementary Fig. 6). As shown in Fig. 7b, the onset potential for PAQ-CO_2_ oxidation (ca. −0.26 V vs. Ag/AgCl) can become more positive than the onset potential for ORR (ca. −0.43 V vs. Ag/AgCl) in 20m LiTFSI, such that the adduct will be much less prone to react with O_2_ in salt-concentrated aqueous media. This is not the case at low salt concentration. Adopting quinones with higher redox potentials can further increase the potential difference for even more stable adducts (Supplementary Fig. 17).

To further investigate the stability of the quinone-CO_2_ adduct, we quantified the degree of PAQ-CO_2_ oxidation in the presence of O_2_. Specifically, the PAQ electrodes were first fully reduced under CO_2_ and were then allowed to rest in the presence of different concentrations of O_2_, after which the electrodes were reduced again to quantify the capacity loss (Fig. 7c and Supplementary Fig. 18). Merely 9.4% of the adducts were oxidized after resting in air for 15 min, which is the typical capture-release time scale for our electro-swing CO_2_ separation; and even when rested in pure O_2_, only 23.2% of the capacity was lost after 15 min. Moreover, in the presence of 3% O_2_, which is the typical concentration in coal-fired power plant flue gas, 90% of the capacity was maintained after 1 h. While further improvements will be required for full practical implementation, these results demonstrate a promising pathway to minimizing the effects of oxygen on quinone capture processes.

Figure 7d shows the CV scans of PAQ under pure CO_2_ and simulated flue gas (15% CO_2_ with 3% O_2_). To compare the efficiency under the two scenarios, we calculated the ratio between the total anodic and cathodic capacities (*Q*_a_/*Q*_c_). At a scan rate of 10 mV s^−1^, the value was 87.3% under pure CO_2_ and decreased slightly to 80.7% under simulated flue gas. At an even slower scan rate of 5 mV s^−1^ to ensure the complete reaction between PAQ dianions and CO_2_, a high *Q*_a_/*Q*_c_ of 83.6% can be achieved (Supplementary Fig. 19a). Replacing PAQ with NQ afforded further improved efficiency (Supplementary Fig. 19b). When constant current CO_2_ capture-release cycling was conducted under simulated flue gas in the membrane flow cell configuration, high capacity cycling with good stability was still realized (Fig. 7e) with only moderately compromised efficiency (the average Coulombic efficiency over 75 cycles was 95.5%, as shown in Supplementary Fig. 20). The electrical energy consumption of this capture-release process was calculated to be ~56 kJ mol^−1^ CO_2_ (Supplementary Note 2), which is on par with other carbon capture systems^44^. A more detailed energetic analysis of quinone-mediated carbon capture in a conventional electrolyte can be found in a recent literature^16^. The process energetics can be further improved with engineering optimization. With the data above, we believe our proposed system of electrochemically mediated carbon capture in salt-concentrated aqueous media can indeed be of practical significance for carbon capture from point sources.

## Discussion

In this work, we investigated the feasibility of employing salt-concentrated aqueous electrolytes for electrochemically mediated CO_2_ separation using quinone chemistry. By rationally designing the electrolyte formulation, salt-concentrated aqueous electrolytes can offer an extended electrochemical stability window, high CO_2_ activity, as well as significantly reduced active material dissolution and evaporative loss. Importantly, reduced quinone species are much less susceptible to competing reactions such as protonation and ion association in salt-concentrated media compared to their dilute counterparts due to the modified dielectric environment, which is highly desirable for quinone-CO_2_ adduct formation. As a result, high capacity CO_2_ capture-release cycling with excellent electrokinetics, efficiency and stability was demonstrated in both conventional single-compartment electrochemical cell and prototypical membrane-based flow cell. When tested using simulated flue gas, our proposed system maintained good performance metrics and competitive system energetics. The proved advantages of salt-concentrated aqueous electrolytes are also transferable to other types of redox-active molecular CO_2_ sorbents. The material design demonstrated through these results advances the practicality of electrochemically mediated carbon capture with redox-active molecular CO_2_ sorbents broadly, given that the inherent advantages are not limited to our example quinone chemistry.

## Methods

### Materials

The electrolytes were prepared by dissolving the corresponding salts, lithium bis(trifluoromethanesulfonyl)imide (Solvay), and/or tetrabutylammonium hexafluorophosphate (Sigma-Aldrich) in water and/or DMSO (Sigma-Aldrich). Ferrocene and anthraquinone were purchased from Sigma-Aldrich.

### Synthesis of the PAQ-CNT electrode

PAQ was synthesized following a previously reported procedure^32^. To obtain the PAQ-CNT composite, PAQ was dissolved in chloroform (1 mg mL^−1^) using a probe sonicator at 5 °C (Cole-Parmer Ultrasonic Processor, pulser mode: on 5 s, off 3 s, 60% amplitude). Subsequently, CNT was added into the solution (1 mg mL^−1^), and the mixture was sonicated for another 20 min to afford a homogeneous dispersion. The PAQ-CNT ink was then cast on either glassy carbon electrode (Ø 3 mm, BASi) or 50 μm carbon felt (Fibre Glast Carbon Fiber Veil).

### Characterizations

SEM images were taken with a Zeiss SUPRA 55-VP scanning electron microscope. XPS analysis was obtained on a Thermo Scientific K-Alpha+ XPS equipped with Al (Kα) source with a spot size of 400 µm. High-resolution spectra were collected with a step size of 0.1 eV and an accumulation of ten scans. Hydroquinone solubility was determined based on UV−Vis absorption spectra (Ocean Optics). The evaporative loss rate of the electrolytes was measured by monitoring their weight change under constant N_2_ purging. In each experiment, 5 mL electrolyte was sealed in a 20-mL glass vial with septum cap, which was equipped with a 21G needle inlet and a 21G needle outlet. Pure N_2_ gas was bubbled directly into the electrolyte through the needle inlet at a flow rate of 200 mL min^−1^.

### CO_2_ capture/release quantification by FTIR spectroscopy

A Nicolet 8700 FTIR spectrometer (Thermo Scientific) and a 100-mm short-path IR gas cell (Pike Technologies, septum-sealed) were employed for the quantification of CO_2_ capture/release by PAQ. The spectrometer chamber was under high-flow-rate N_2_ purging during the measurements to avoid interference from atmospheric CO_2_. The calibration curve was made by injecting (Hamilton 1700 series gastight syringes with 30G needle) defined amounts of pure CO_2_ into the N_2_ flushed IR gas cell and measuring the integrated area of the two CO_2_ asymmetric stretching bands in absorbance mode (2335 and 2360 cm^−1^). For the quantification, CO_2_ was captured electrochemically by PAQ (0.25 mg on carbon felt) via two CV reduction cycles at a slow scan rate of 5 mV s^−1^. The electrode was then removed from the electrochemical cell, thoroughly dried with paper towel, and sealed in a 2-mL N_2_ flushed glass vial with septum cap (Agilent autosampler vial). Thermal release instead of electrochemical release was chosen to avoid the outgassing of physically dissolved CO_2_ from the electrolyte in the case of electrochemical release, which can compromise the quantification accuracy. After 30-min thermal release on a hotplate, 250 μL headspace gas was taken and injected into the N_2_ flushed IR gas cell to obtain the corresponding IR spectrum. The capture and release experiments were done in triplets at each thermal release temperature.

### Electrochemical measurements

All the electrochemical measurements were conducted using a VersaSTAT4 potentiostat (Princeton Applied Research) in a conventional single-compartment electrolytic cell (BASi), unless otherwise specified. The glassy carbon electrode used in this study has a diameter of 3 mm and was polished before each measurement. The LiFePO_4_ on Al foil electrode was purchased from MTI Corp. (single side coated, active material loading 12 g cm^−2^). The LiFePO_4_ on carbon felt electrode was prepared by mixing 95% LFP (MTI Corp.), 2.5% carbon black (Super P, MTI Corp.) and 2.5% polyvinylidene fluoride (Sigma-Aldrich) in *N*-methylpyrrolidinone followed by casting on 50 μm carbon felt (mass loading 8 mg cm^−2^). For the construction of the PAQ-LiFePO_4_ membrane flow cell, PAQ-CNT was immobilized on 50 μm carbon felt at a mass loading of 1 mg cm^−2^ and LiFePO_4_ on Al foil was used as the counter electrode. Both electrodes were 2 cm × 2 cm in area and were separated by an electrolyte-imbibed glass fiber membrane (MilliporeSigma). A hydrophobic gas diffusion layer (Toray carbon paper, PTFE treated, TGP-H-60) was placed on one side of the PAQ electrode to interface with continuous CO_2_ flow. The reduction of PAQ was conducted with a cut-off voltage of either −0.95 V vs. Ag/AgCl or −1.4 V vs. LiFePO_4_ counter electrode to prevent the possible TFSI^−^ decomposition. The oxidation of PAQ-CO_2_ was conducted via the CC/CV mode (constant current followed by constant voltage). The cut-off voltage for CC oxidation was either 0.2 V vs. Ag/AgCl or −0.1 V vs LiFePO_4_; CV oxidation was carried out until the current decayed to 10% of the CC oxidation current.

### In situ gas cell measurements

Measurements on the CO_2_ permeation response during quinone cycling were conducted using a house-machined device consisting of two gas flow chambers separated by the PAQ-LiFePO_4_ electrochemical cell, all held together with rubber gaskets and screws (detailed gas cell configuration is shown as Supplementary Fig. 16). Both PAQ-CNT (1 mg cm^−2^) and LiFePO_4_ (8 mg cm^−2^) were cast on 50 μm carbon felt, and the electrode area was 2 cm × 2 cm. The two electrodes were separated with a 25 μm polypropylene separator (Celgard 3501) infused with 20m LiTFSI electrolyte. The PAQ-LiFePO_4_ electrochemical cell was mounted on a brass gasket holder with a 1 cm^2^ circular opening, and the edges of the electrochemical cell were sealed to block gas-phase CO_2_ transport between the two flow chambers. The brass gasket also served as the current collector for the PAQ-CNT electrode, and a titanium mesh was used as the current collector for the LiFePO_4_ electrode. The testing employed a “zero-gap” design where the electrochemical components were all in close contact, such that CO_2_ cross-over can only occur via dissolution-diffusion through the electrolyte. During operation, CO_2_ flowed through one gas chamber and N_2_ sweep gas flowed through the other. The concentration of CO_2_ in the N_2_ stream was measured with an in-line CO_2_ sensor (ExplorIR^®^-W 20% CO_2_ sensor). The sensor has a measurement range of 0−20% CO_2_ and an accuracy of ±70 ppm. The electrochemical cycling followed a constant voltage protocol. For each capture-release cycle, the cell was first held at −1.2 V for 10 min to capture CO_2_, followed by release at 0 V for 10 min and a 10-min rest.

## Supplementary information

Supplementary Information

## Data Availability

The datasets generated during and/or analyzed during the current study are available from the corresponding author on reasonable request. The source data underlying Figs. 2a, c, d, 3a, d−f, 4a, c−e, 5a, b, d, 6b, 7a−e and Supplementary Figs. 17, 19, 20 and 22 are provided as a Source Data file.

## Source data

Source Data
