# Supplementary material for: Electrochemically mediated carbon dioxide separation with quinone chemistry in salt-concentrated aqueous media
Source: Nat Commun. 2020 May 8;11:2278. doi: 10.1038/s41467-020-16150-7 (PMC7211026; doi:10.1038/s41467-020-16150-7)
Supplement: Supplementary file 1 — Supplementary Information [file 41467_2020_16150_MOESM1_ESM.pdf]

Supplementary Information

Electrochemically mediated carbon dioxide separation with quinone chemistry in salt-concentrated aqueous media

Liu et al.

Supplementary Figures

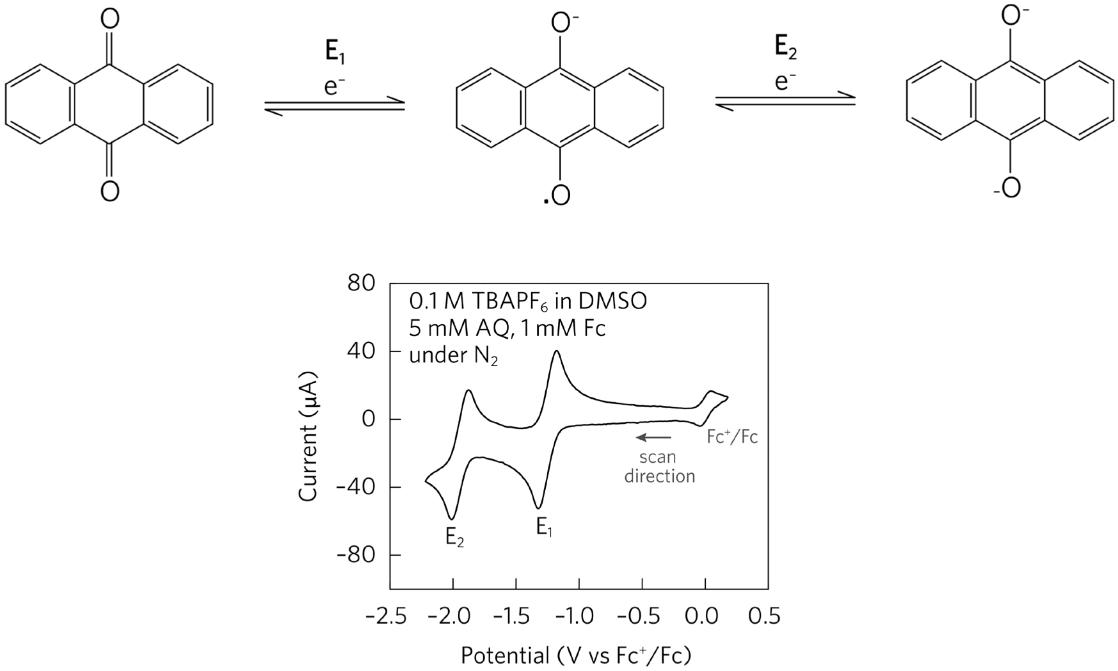

Supplementary Figure 1. Two one–electron reduction waves of anthraquinone (AQ) in the absence of electrophiles in aprotic solvents. The CV was carried out in 5 mM AQ in dimethyl sulfoxide (DMSO) with 0.1 M tetrabutylammonium hexafluorophosphate ($TBAPF_6$) supporting salt under N_2 atmosphere. Glassy carbon was used as the working electrode, Pt wire was used as the counter electrode, Ag wire was used as a pseudo–reference electrode, and 1 mM ferrocene (Fc) was used as an internal reference. The scan rate was 50 mV s^{-1}.

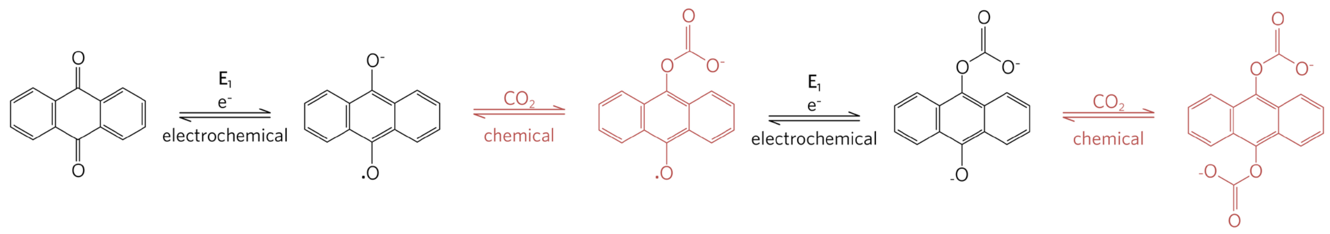

Supplementary Figure 2. Reaction steps of electrochemically mediated carbon capture with anthraquinone. Complexation of CO_2 with quinone following an ECEC mechanism. The electrochemical steps are in black and the chemical steps are in red.

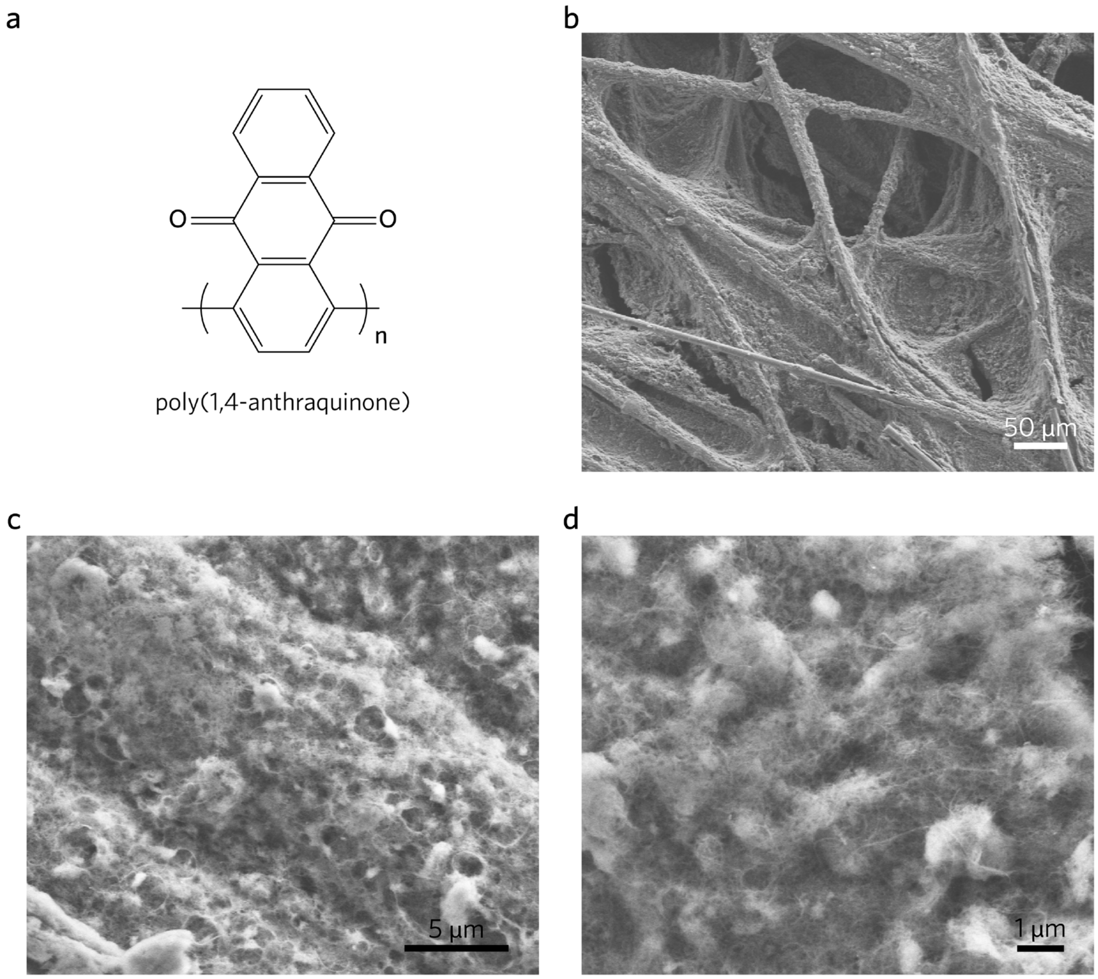

Supplementary Figure 3. Characterization of the PAQ electrode. (a) Molecular structure of PAQ, and (b–d) scanning electron microscopy images of PAQ–CNT drop–cast on carbon felt substrate used in this study.

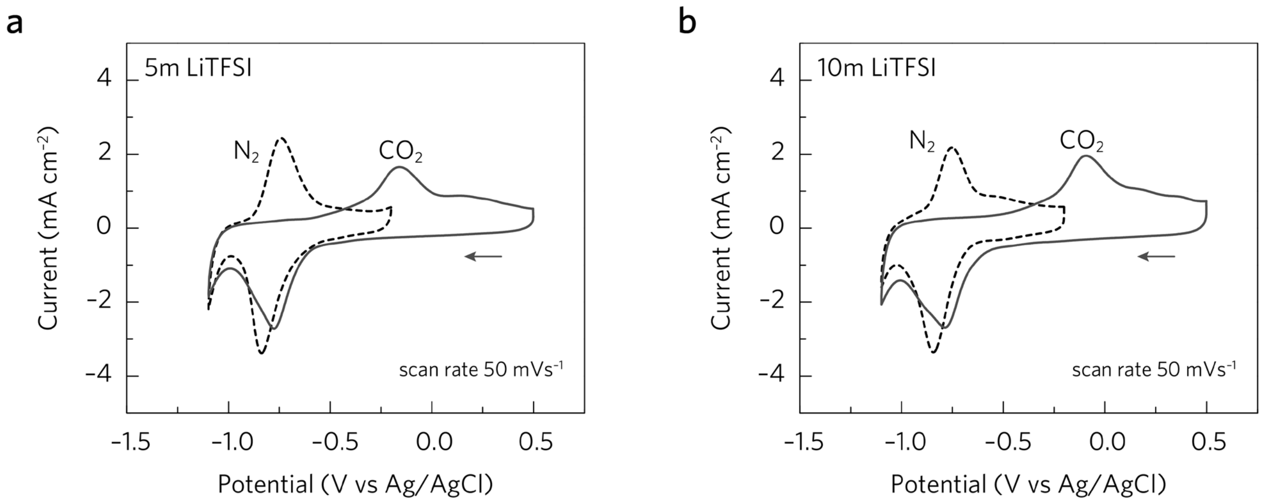

Supplementary Figure 4. Additional CV scans in aqueous electrolytes of different LiTFSI concentrations. CV of PAQ–CNT in (a) 5m LiTFSI, and (b) 10m LiTFSI, under N_2 (dashed line) and CO_2 (solid line). PAQ was cast on glassy carbon at a loading of 0.042 mg cm^{-2}. The scans were conducted at 50 mV s^{-1}, with Pt wire as the counter electrode and Ag/AgCl as the reference electrode.

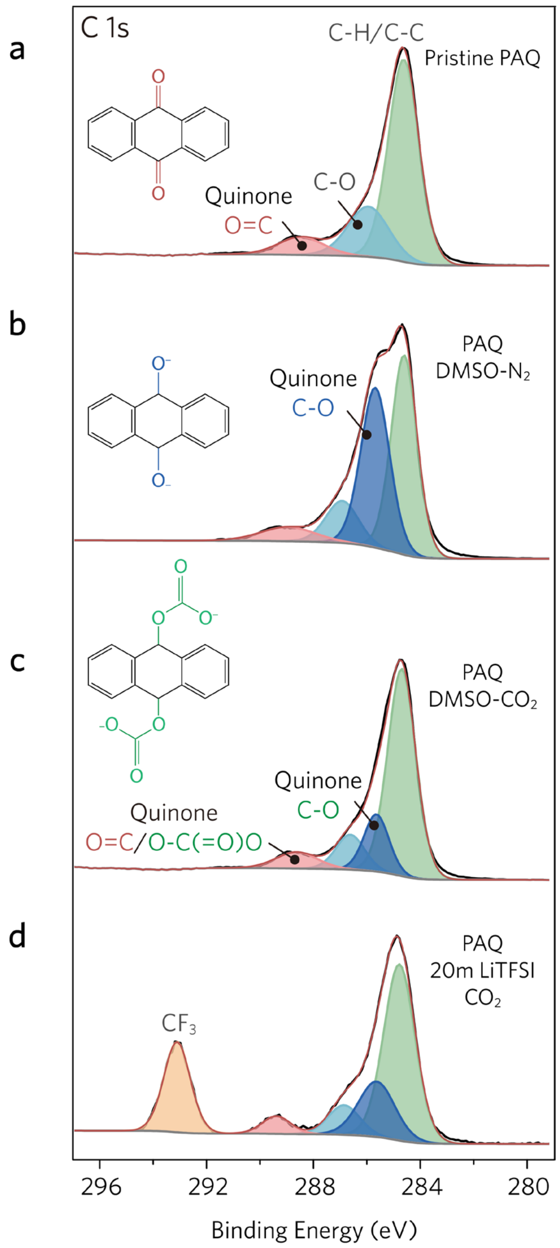

Supplementary Figure 5. XPS characterization of the PAQ electrodes. XPS high–resolution C1s spectra of (a) pristine PAQ, electrochemically reduced PAQ in DMSO–based electrolyte (0.1M $TBAPF_6$ in DMSO) (b) without and (c) with CO_2, and (d) electrochemically reduced PAQ in 20m LiTFSI with CO_2. Aromatic & adventitious C–C, C–H are assigned at 284.6 eV (green peak), adventitious C–O (also from CNT) is assigned at 286.6 eV (light blue peak), C–O from reduced quinone is assigned at 285.7 eV (dark blue peak), quinone carbonyl C=O and carbonate from quinone–CO_2 adduct are assigned as 288.5–289 eV (pink peak)[1]. The CF_3 peak in (d) is attributed to the residue LiTFSI salt on the electrode surface. The spectrum of PAQ reduced in 20m LiTFSI under CO_2 showed a close resemblance to that of PAQ–CO_2 adduct obtained using conventional DMSO–based electrolyte, confirming the successful adduct formation in salt–concentrated aqueous electrolyte.

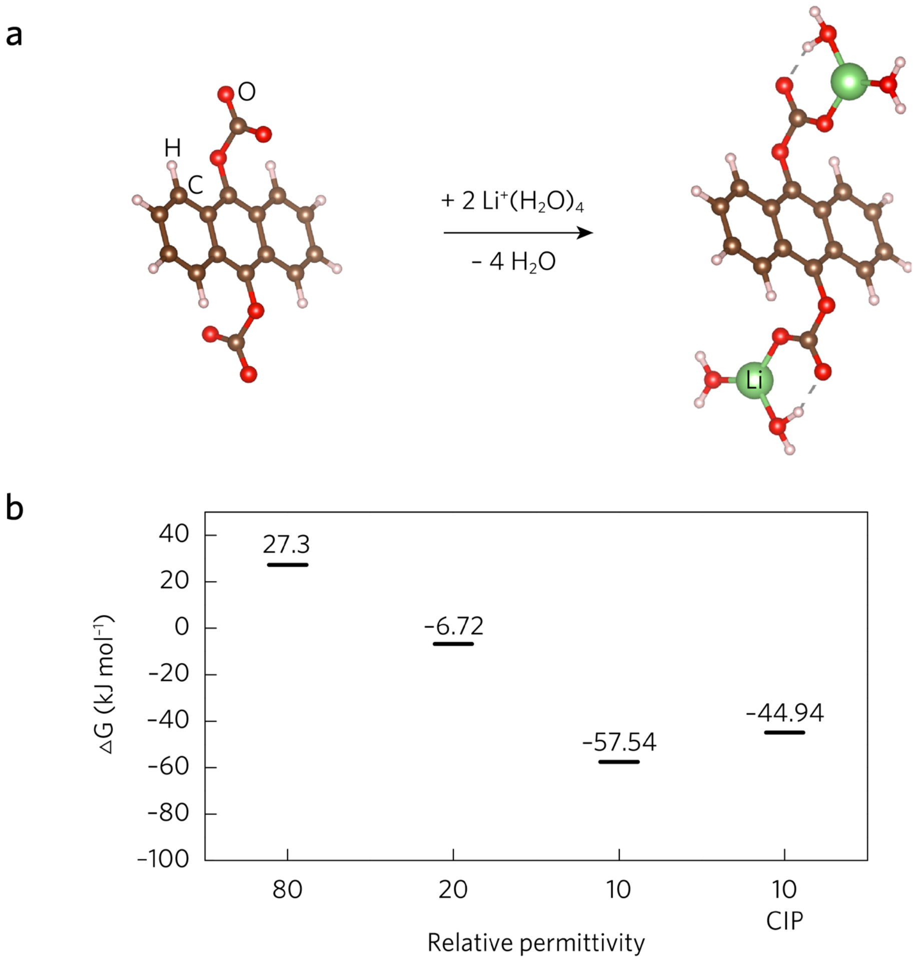

Supplementary Figure 6. DFT calculation of the complexation between quinone bis(carbonate) anion and Li^+. (a) Illustration of the complexation process between quinone bis(carbonate) anion and Li^+. Each free Li^+ is solvated by four H_2O molecules and each coordinated Li^+ is solvated by two H_2O molecules, which are determined as the most stable configurations. (b) The free energy change for the complexation between quinone bis(carbonate) and Li^+ at different electrolyte permittivity. The complex becomes more stable at higher LiTFSI concentration. A permittivity of 80 and 10 corresponds to 1m and 20m LiTFSI, respectively. CIP corresponds to the complexation between quinone bis(carbonate) and LiTFSI CIP solvated by two H_2O molecules.

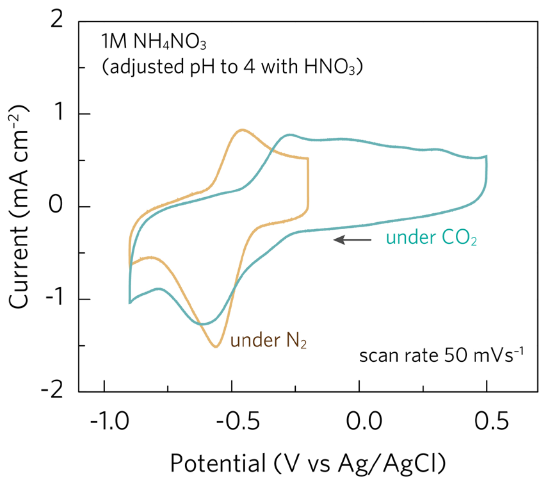

Supplementary Figure 7. CV of PAQ–CNT in 1m NH_4NO_3 (pH=4) under N_2 and CO_2. PAQ was cast on glassy carbon at a loading of 0.042 mg cm^{-2}. The scans were conducted at 50 mV s^{-1}, with Pt wire as the counter electrode and Ag/AgCl as the reference electrode.

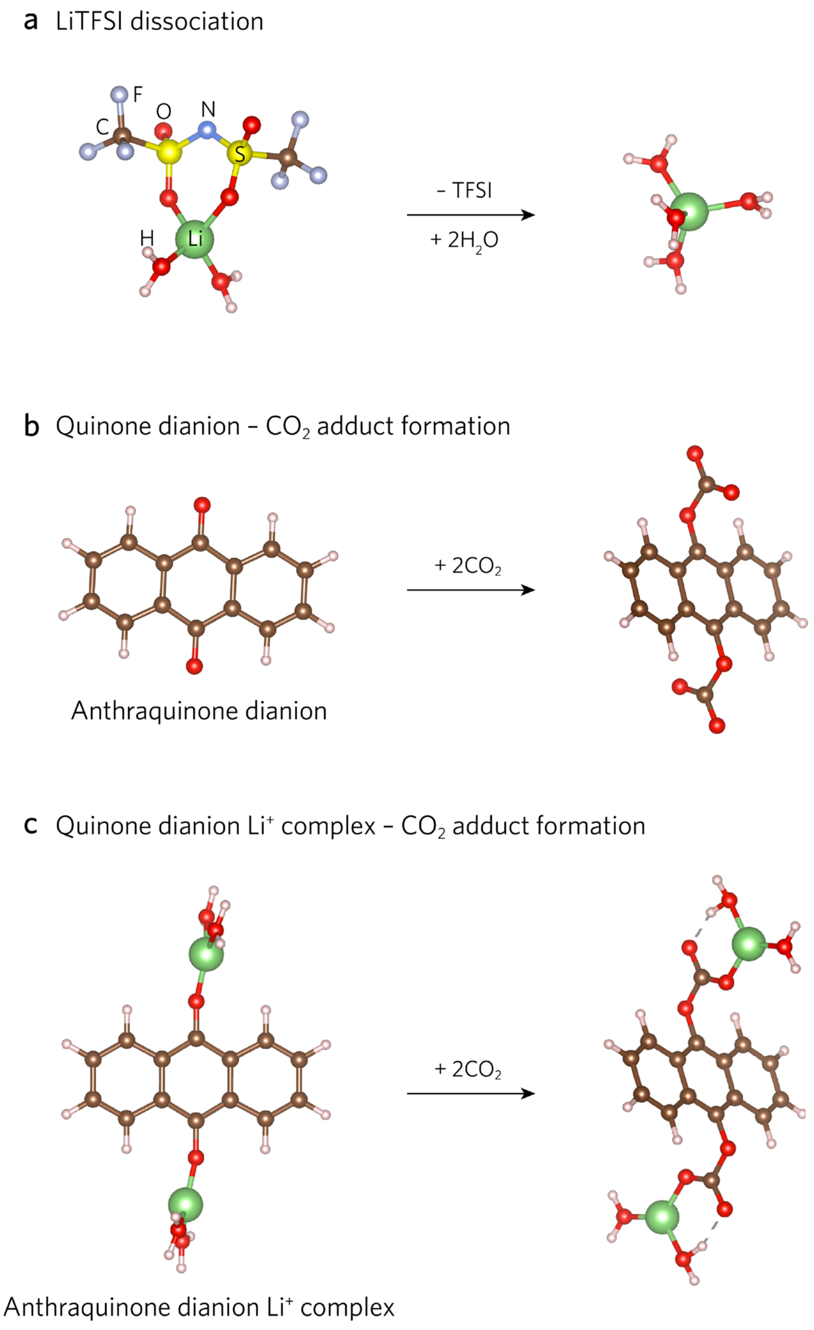

Supplementary Figure 8. Diagrams illustrating the detailed processes calculated using DFT. (a) LiTFSI dissociation, which is the process plotted in Figure 3b. Each associated LiTFSI is solvated by two H_2O molecules and each free Li^+ is solvated by four H_2O molecules, which are determined as the most stable configurations. The driving force (ΔG) of this process in different dielectric environments are (in kJ/mol): –23.52 (ε=80), –11.34 (ε=20), +6.30 (ε=10). (b) Formation of quinone–CO_2 adduct from quinone dianion, which is the "$AQ^{2-} \rightarrow$ adduct" process plotted in Figure 3c. The driving force (ΔG) of this process in different dielectric environments are (in kJ/mol): –45.78 (ε=80), –47.46 (ε=20), –49.98 (ε=10). (c) Formation of quinone–CO_2 adduct from quinone dianion complexed with Li^+, which is the "$AQ^{2-} + 2Li^+ \rightarrow$ adduct" process plotted in Figure 3c. The driving force (ΔG) of this process in different dielectric environments are (in kJ/mol): –12.18 (ε=80), –15.96 (ε=20), –18.48 (ε=10).

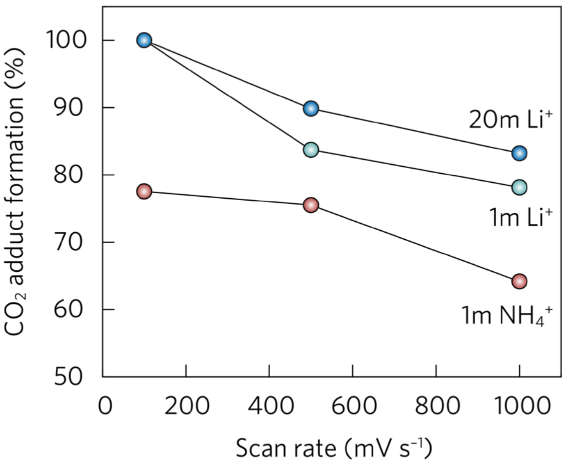

Supplementary Figure 9. The percentage of quinone–CO_2 adduct formation as a function of CV scan rates in 1m NH_4NO_3, 1m LiTFSI and 20m LiTFSI electrolytes. The percentage was calculated by integrating and comparing the oxidation peaks of quinone–CO_2 adduct and quinone dianions, based on data presented in Figure 3d–f. Percentage of adduct formation = adduct oxidation peak area/(adduct oxidation peak area + quinone dianion oxidation peak area).

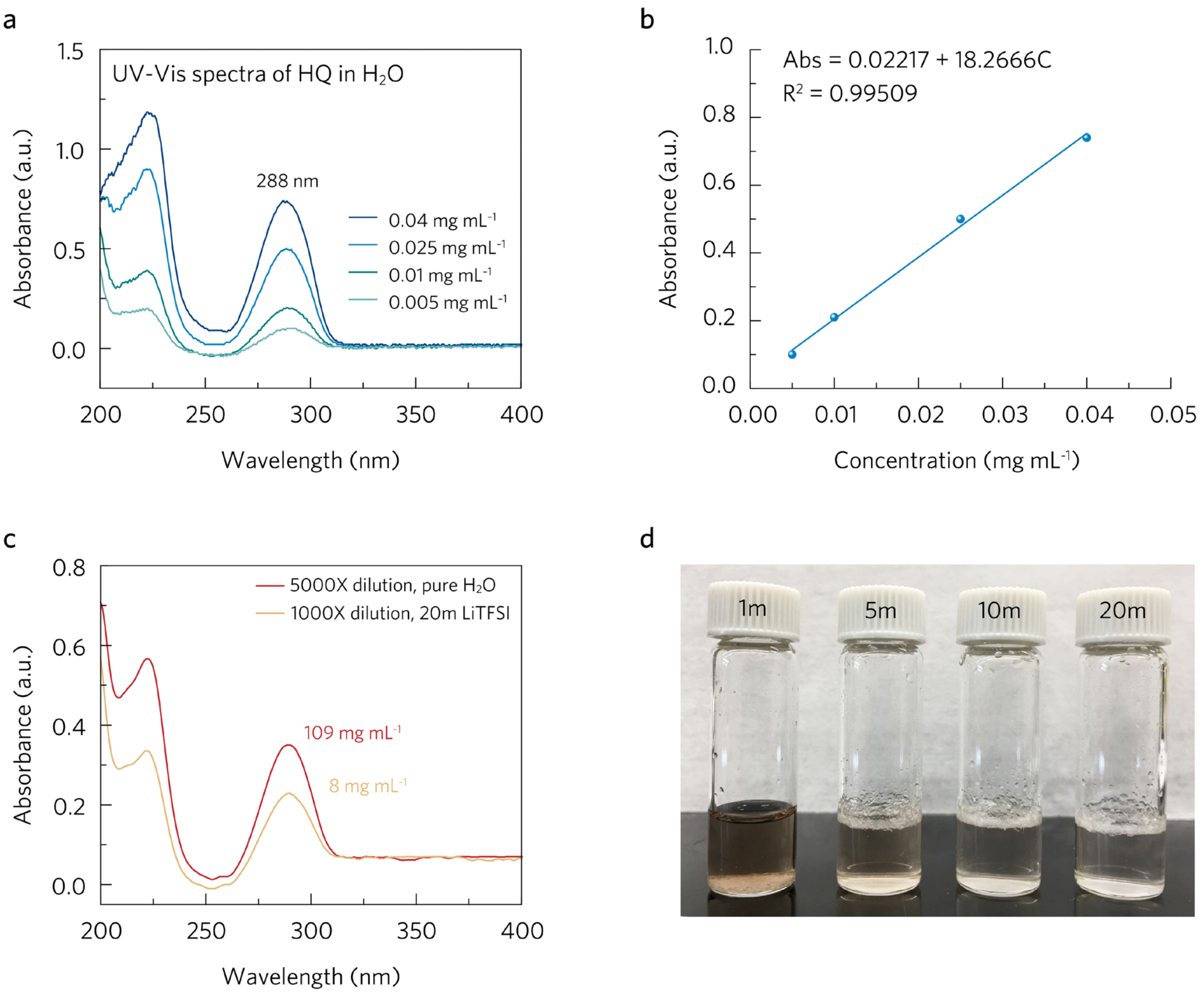

Supplementary Figure 10. Hydroquinone (HQ) solubility in aqueous media. (a) UV–Vis absorption spectra of different concentrations of HQ aqueous solutions, and (b) the corresponding calibration curve (absorbance at 288 nm). (c) UV–Vis absorption spectra of saturated HQ in pure water (diluted by 5,000 times) and in 20m LiTFSI electrolyte (diluted by 1,000 times). The solubility of HQ was determined to be 109 mg mL^{-1} in pure water and 8 mg mL^{-1} in 20m LiTFSI. (d) Photo image of saturated HQ in electrolytes of different concentrations (1m, 5m, 10m, and 20m LiTFSI). The oxidative polymerization of HQ in water resulted in brown–colored oligomers which serves as a direct visual indication of HQ solubility. The photo was taken 2 hours after HQ dissolution.

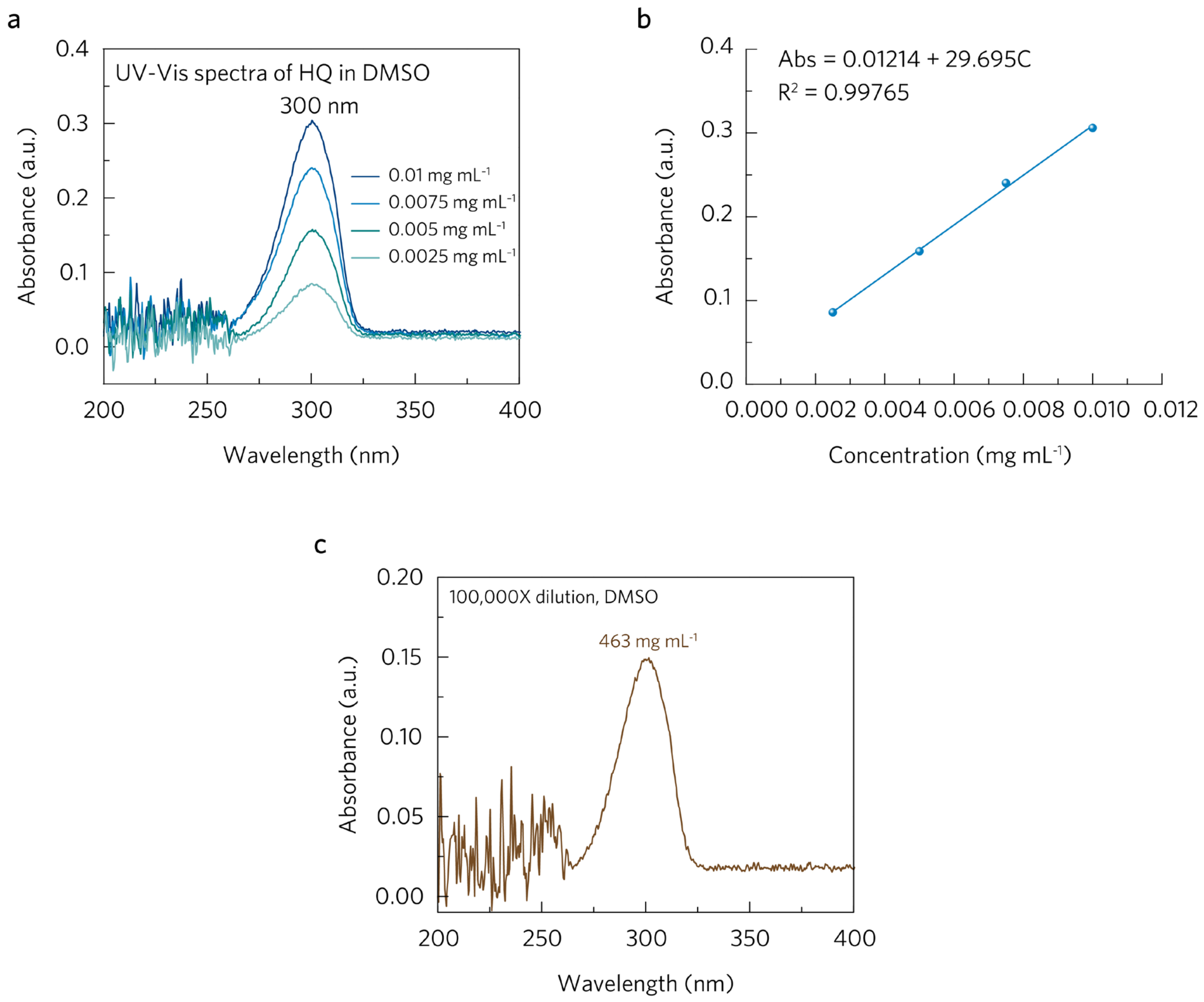

Supplementary Figure 11. HQ solubility in DMSO. (a) UV–Vis absorption spectra of different concentrations of HQ in DMSO, and (b) the corresponding calibration curve (absorbance at 300 nm). (c) UV–Vis absorption spectrum of saturated HQ in DMSO diluted by 100,000 times. The solubility of HQ in DMSO was determined to be 463 mg mL^{-1}.

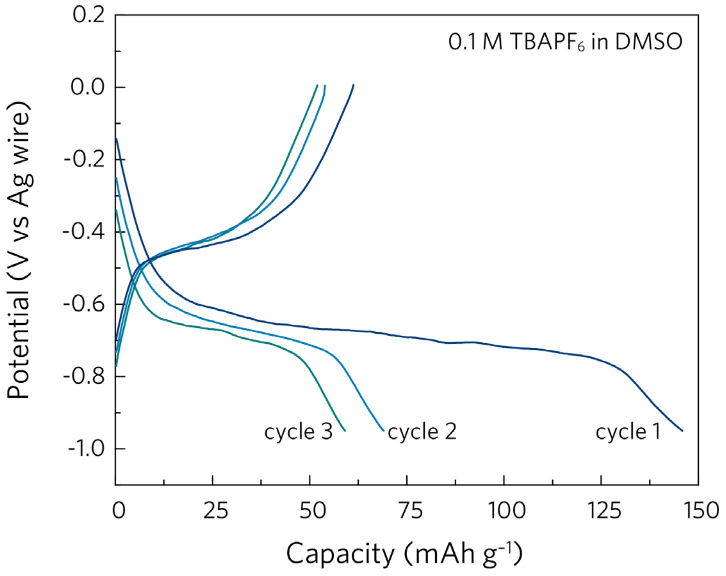

Supplementary Figure 12. Charge–discharge voltage profiles of PAQ–CNT immobilized on carbon felt electrode in DMSO–based electrolyte (0.1M $TBAPF_6$ in DMSO). The PAQ mass loading was 0.1 mg cm^{-2}, Ag wire was used as the reference electrode and Pt wire was used as the counter electrode. The cycling was conducted at a current density of 0.2 mA cm^{-2}.

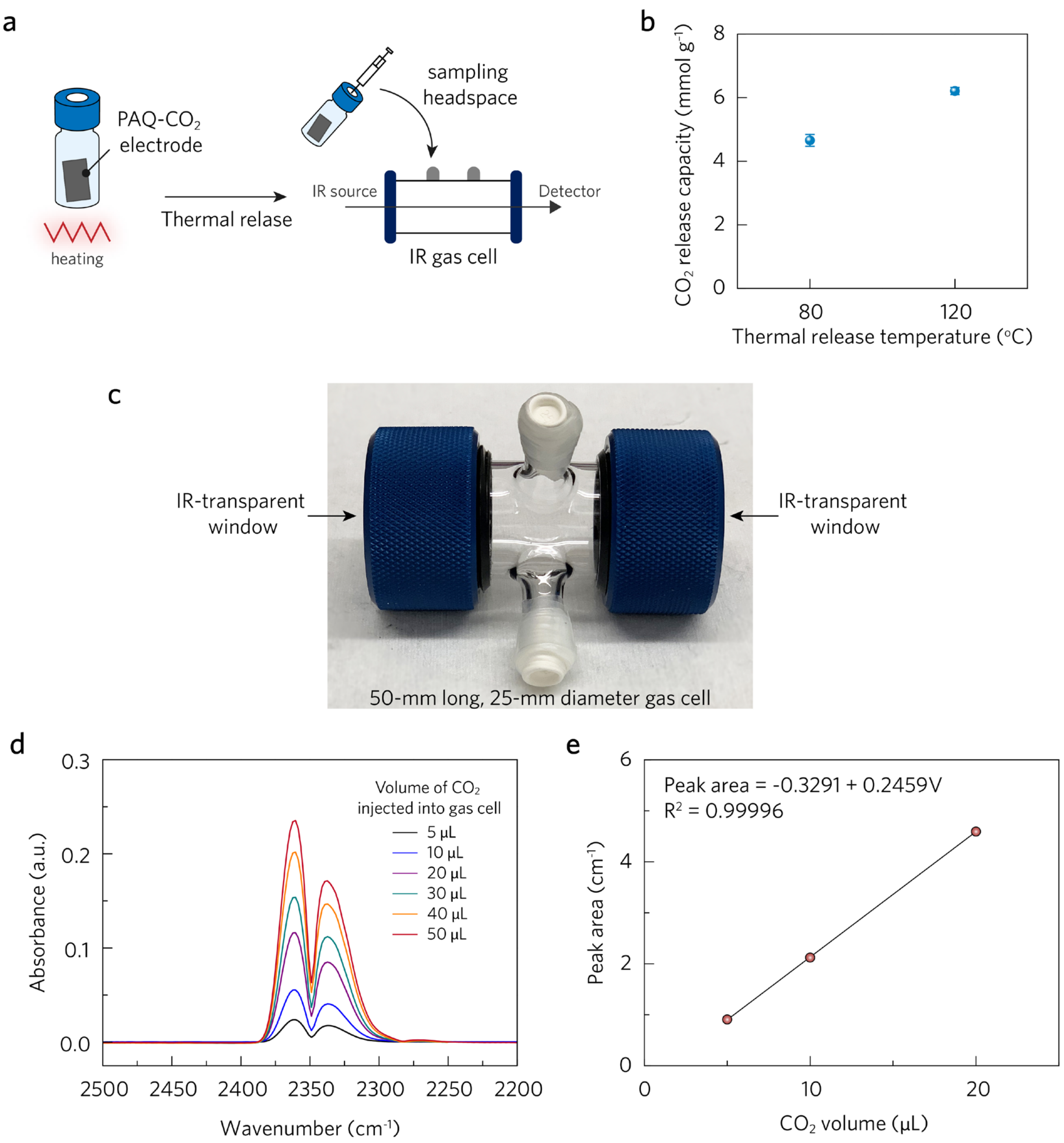

Supplementary Figure 13. Quantification of CO_2 uptake by the PAQ electrode using FTIR gas cell. (a) Schematic illustrating the experimental procedure. PAQ–CNT immobilized on carbon felt electrode was first reduced electrochemically in 20m LiTFSI to form the PAQ–CO_2 adduct. The electrode was then placed in a sealed vial to thermally release the captured CO_2, followed by sampling the headspace gas with an FTIR gas cell to determine the amount of released CO_2. (b) The CO_2 thermal release capacity at two release temperatures determined by FTIR. (c) Photo image of the gas cell. The two gas sampling ports were sealed with rubber septa. The gas cell was flushed with N_2 before each measurement, and gas samples were injected into the gas cell through the septa using Hamilton gas–tight syringe equipped with 30G needle. (d) IR spectra of different amounts of CO_2 injected into the gas cell, and (e) the corresponding calibration curve based on the integrated peak area of the characteristic asymmetric stretching bands of gaseous CO_2.

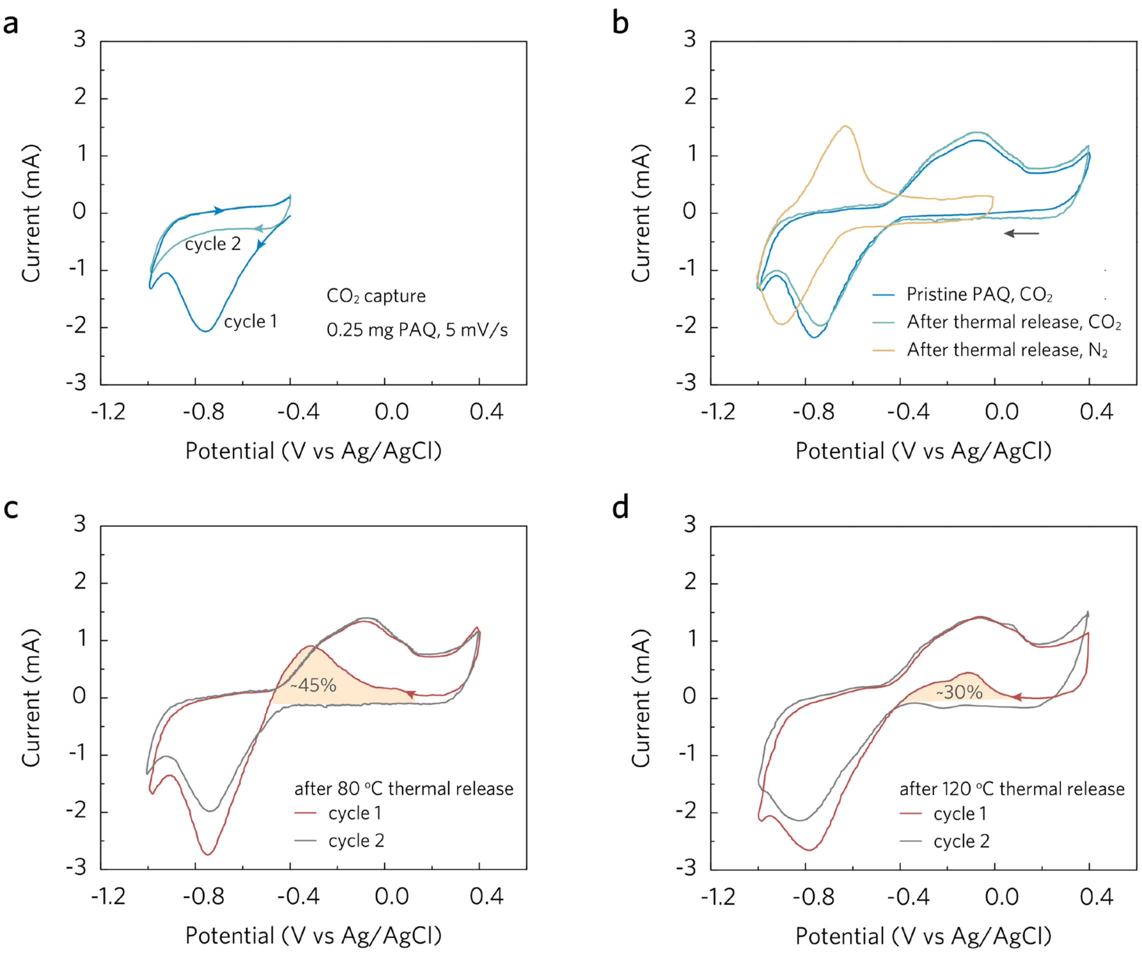

Supplementary Figure 14. Electrochemical CO_2 capture followed by thermal release for CO_2 uptake quantification using FTIR gas cell. (a) CO_2 was captured by PAQ electrochemically via two CV reduction cycles at a scan rate of 5 mV s^{-1}. No reduction peak can be observed during the second CV scan, confirming the complete formation of PAQ–CO_2 adducts. (b) CV scans of the PAQ–CNT electrode before thermal release (under CO_2) and after thermal release (under both N_2 and CO_2). The thermally–regenerated electrode showed similar electrochemical behavior compared to its pristine state, indicating the good reversibility and stability of the PAQ–CNT electrode through the thermal release process. First two CV cycles of the PAQ–CNT electrode after thermal release at (c) 80 ºC and (d) 120 ºC (under CO_2). An obvious electrochemical oxidation peak can be observed for both cases at the beginning of the first CV cycle, indicating that the electrochemically captured CO_2 was not fully released by the thermal process. Approximately 45% and 30% of the captured CO_2 was not released thermally at 80 ºC and 120 ºC, respectively.

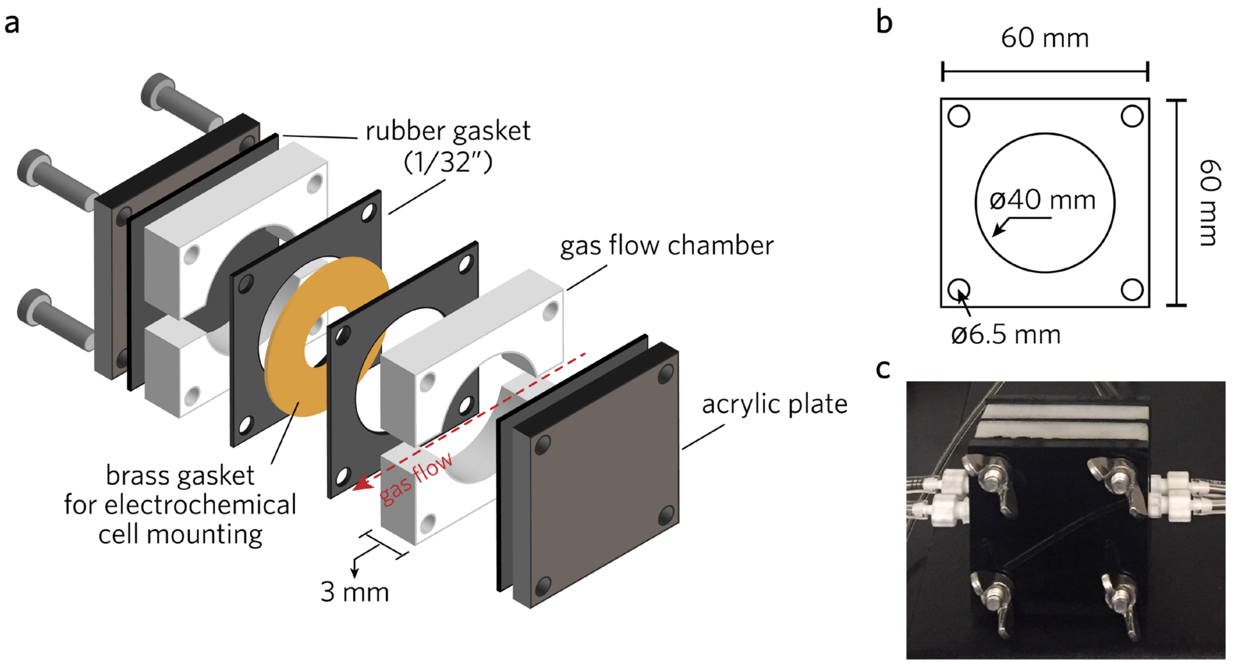

Supplementary Figure 15. Gas cell configuration. (a) Schematic showing the configuration of the gas cell device used in this study. The PAQ–$LiFePO_4$ electrochemical cell was mounted on the brass sample holder disc and the edges of the electrodes were sealed with tape to block gas–phase CO_2 leakage. (b) Dimensions of the gas flow chamber. (c) Photo image of the gas cell device.

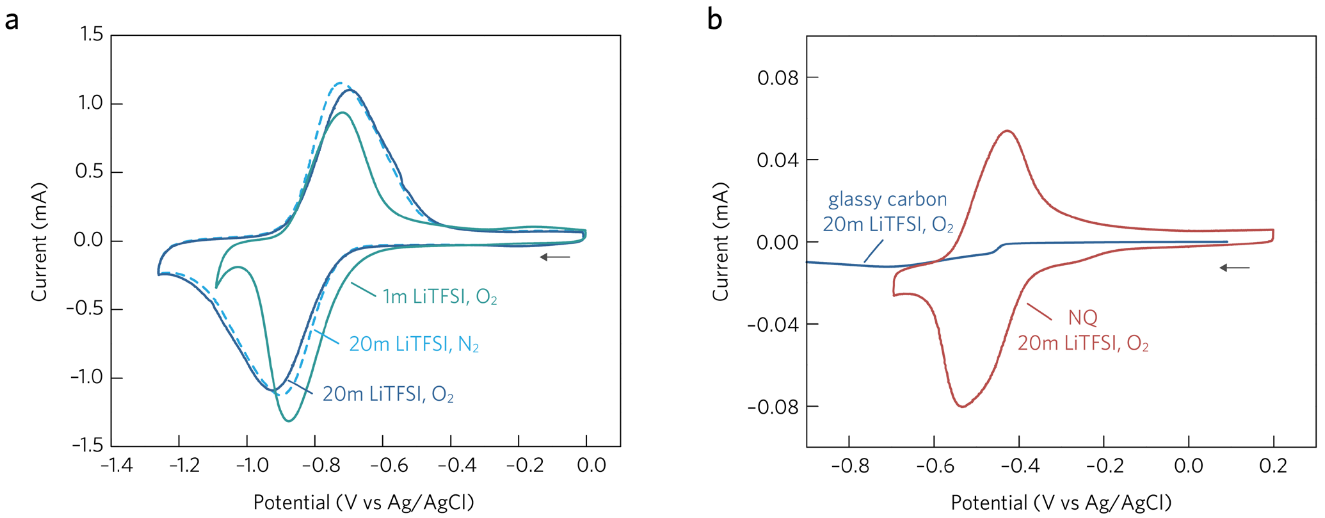

Supplementary Figure 16. Cyclic voltammograms of quinone in the presence of oxygen. (a) CV scans of PAQ–CNT on glassy carbon in 1m and 20m LiTFSI under N_2 and O_2. The ratio between the anodic peak current and the cathodic peak current is 0.98 and 0.97 for 20m LiTFSI under N_2 and O_2 respectively, indicating the relatively low sensitivity of PAQ towards O_2 in 20m LiTFSI. The value decreased to 0.68 under O_2 in 1m LiTFSI, probably due to the higher tendency for oxygen reduction. (b) Comparison between the reduction potential of NQ and molecular oxygen in 20m LiTFSI. The onset potential for NQ reduction falls more positive than oxygen reduction in 20m LiTFSI. For the CV measurements, either 6 μg of PAQ or 3 μg of NQ was loaded on the glassy carbon electrode and the scans were conducted at a rate of 50 mV s^{-1}.

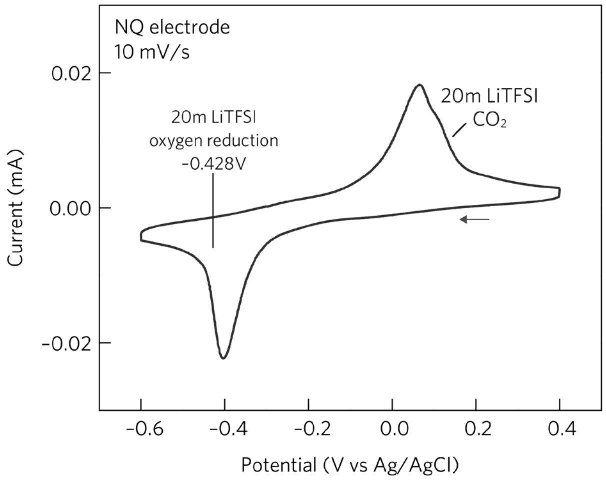

Supplementary Figure 17. Carbon capture–release by naphthoquinone (NQ). CV of NQ under CO_2 in 20m LiTFSI at a scan rate of 10 mV s^{-1} and compared with the oxygen reduction potential. 3 μg of NQ was loaded on the glassy carbon electrode.

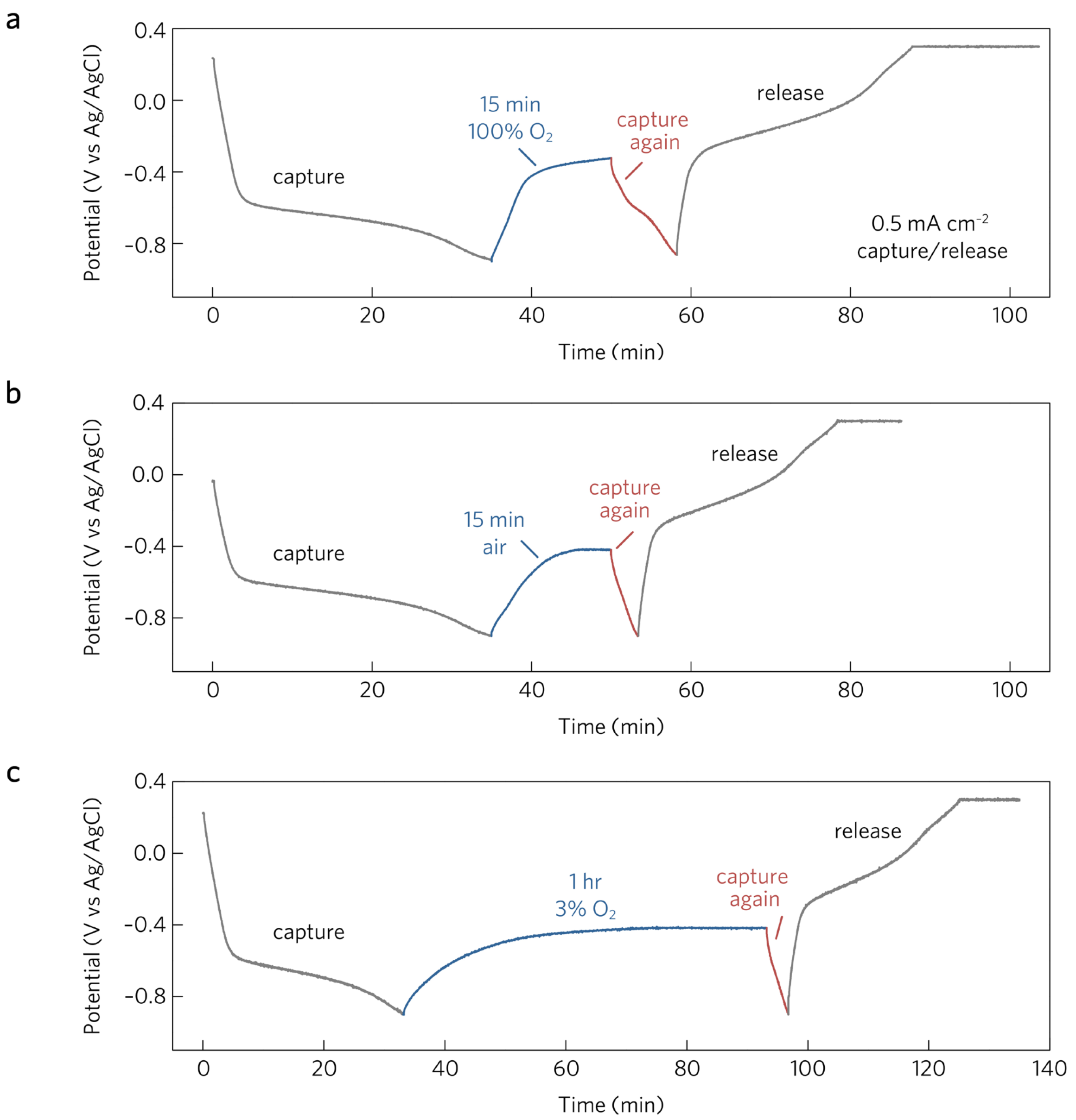

Supplementary Figure 18. Electrochemical tests on the stability of PAQ–CO_2 in the presence of O_2. The PAQ electrodes (immobilized on carbon felt) were first electrochemically reduced under CO_2 to form PAQ–CO_2 adducts, then left to rest under **(a)** pure O_2, **(b)** air, and **(c)** 3% O_2 (typical concentration in coal–fired power plant flue gas), and finally put under CO_2 to be reduced and oxidized again at the same current density (0.5 mA cm^{-2}). The amount of PAQ–CO_2 being oxidized can be quantified from the capacity of the second reduction step (the segment marked in red).

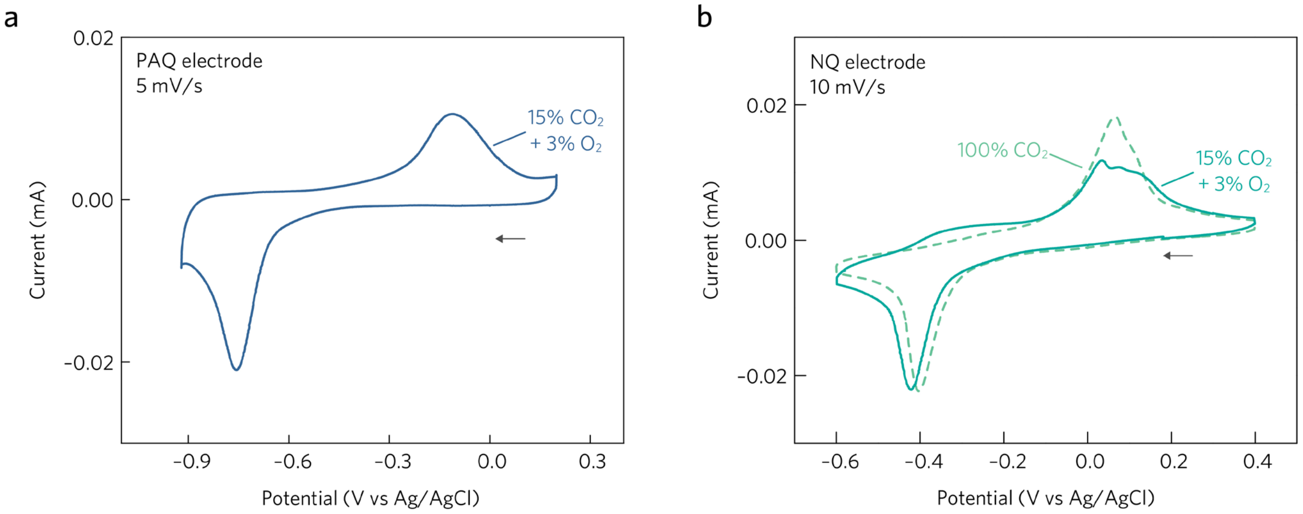

Supplementary Figure 19. Cyclic voltammograms of quinone under simulated flue gas. (a) CV of PAQ under simulated flue gas in 20m LiTFSI at a rate of 5 mV s^{-1}. The ratio between the total anodic capacity and the total cathodic capacity was 83.6%. 1.5 µg of PAQ was loaded on the glassy carbon electrode. (b) CV scans of NQ under 100% CO_2 and simulated flue gas in 20m LiTFSI at a rate of 10 mV s^{-1}. The ratio between the total anodic capacity and the total cathodic capacity was 98.4% and 95.4% for 100% CO_2 and simulated flue gas, respectively. 3 µg of NQ was loaded on the glassy carbon electrode.

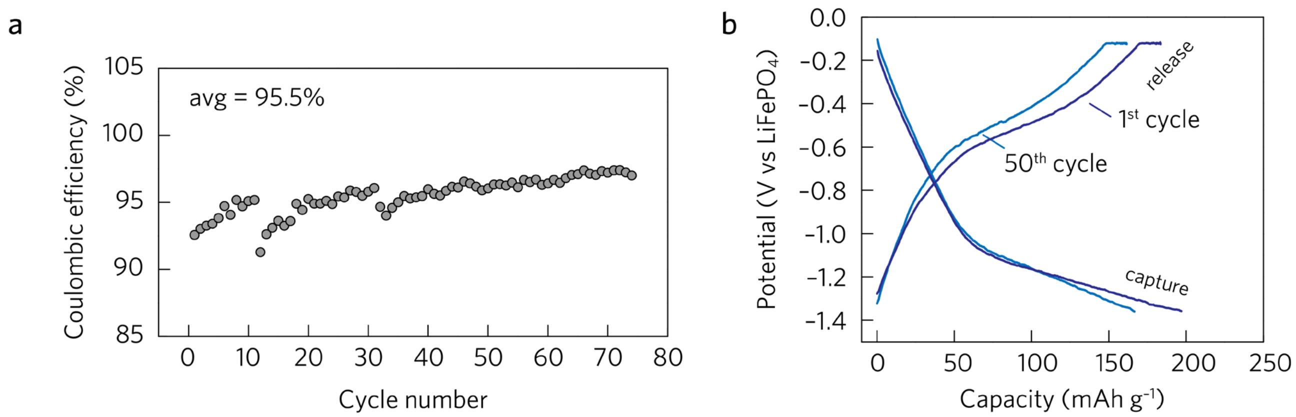

Supplementary Figure 20. Long–term carbon capture–release performance under simulated flue gas. (a) Coulombic efficiency of the PAQ electrode during electrochemical cycling in 20m LiTFSI under simulated flue gas (cycling capacity profile shown in Figure 7e). (b) Voltage profiles of the 1st and the 50th capture–release cycle. The cycling was carried out at a current density of 0.5 mA cm^{-2} and a PAQ mass loading of 0.5 mg cm^{-2} (immobilized on carbon felt).

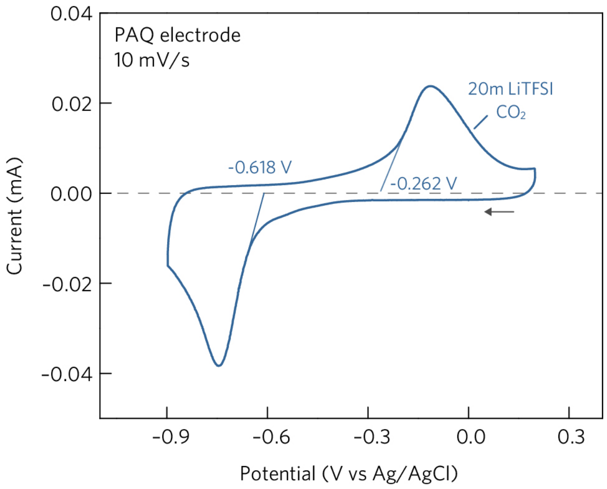

Supplementary Figure 21. Experimental data used for the estimation of the thermodynamic energy consumption. CV of PAQ in 20m LiTFSI under CO_2 at a scan rate of 10 mV s^{-1} with the onset potentials for PAQ reduction and PAQ–CO_2 oxidation labeled.

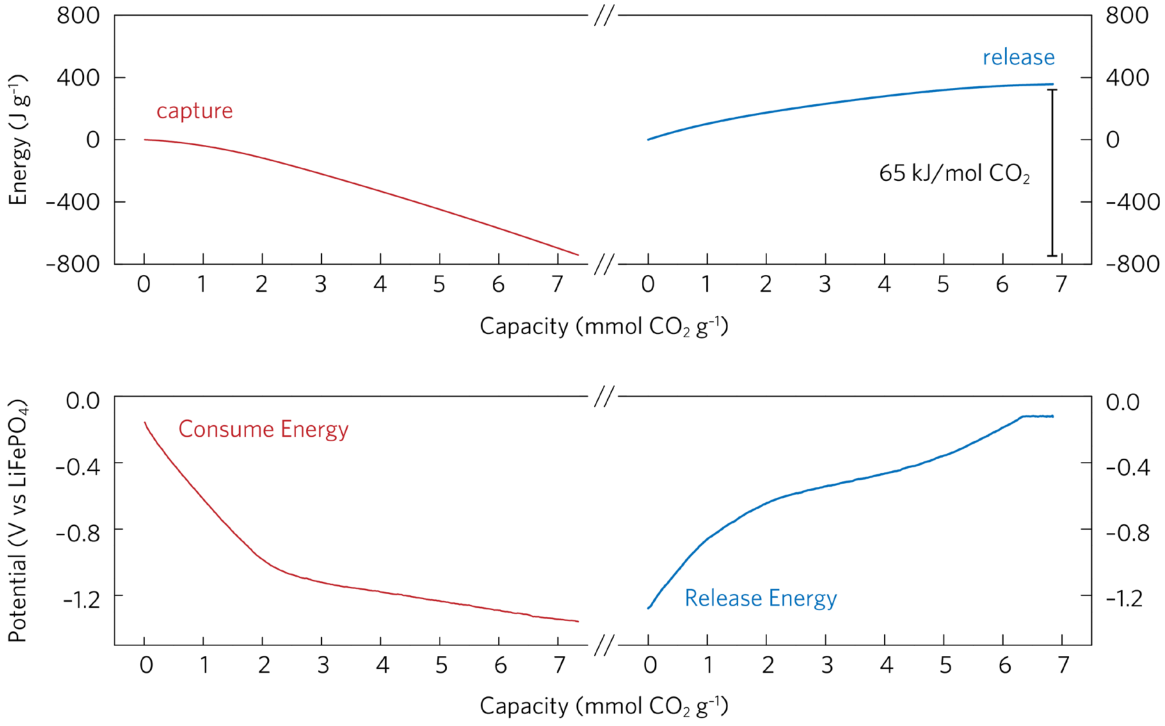

Supplementary Figure 22. Energy consumption in one capture–release cycle under simulated flue gas. If $LiFePO_4$ is used as the counter electrode, the system will consume energy when capturing CO_2 and output energy when releasing CO_2. We assume the energy during release can be recovered.

Supplementary Tables

Supplementary Table 1. Energy change of $Q^{2-} + 2CO_2 \rightarrow Q(CO_2)_2^{2-}$ (Q^{2-} = anthraquinone dianion) predicted by DFT using several XC functionals and DLPNO–CCSD(T) with different values of solvent relative permittivity (at PCM level). All geometries are optimized using the B3LYP XC functional and the 6–31+G** basis except for DLPNO–CCSD(T), for which we use the aug–cc–pVDZ basis.

**Method**	$\epsilon = 80$	$\epsilon = 20$	$\epsilon = 10$	$\epsilon = 1$ **(gas)**
B3LYP/6–31+G**	–34.5	–34.8	–35.4	–44.0
B3LYP+D3/6–31+G**	–41.5	–41.9	–42.4	–50.8
PBE/6–31+G**	–32.4	–32.8	–33.4	–42.6
PBE+D3/6–31+G**	–37.1	–37.5	–38.0	–47.2
wB97x–V/6–31+G**	–50.5	–50.9	–51.5	–60.1
**DLPNO–CCSD(T)/aug–cc–pVDZ**	**–38.5**	**–39.9**	**–40.4**	**–48.0**

Supplementary Notes

Supplementary Note 1. Density functional theory calculations

Density functional theory (DFT) as implemented in Q–Chem is used to obtain the computational results presented in the main text[2]. To determine an exchange–correlation (XC) functional that is appropriate for our system, the energy change of CO_2 captured by hydroquinone anion predicted by various DFT XC functionals is compared to the results obtained using a high–level quantum chemical method, DLPNO–CCSD(T)[3], as implemented in ORCA (Supplementary Table 1)[4]. Following this procedure, geometry optimization and vibrational analysis are performed at the B3LYP/6–31+G** level[5–8], while a single–point energy evaluation at the optimized structure is performed at the PBE+D3/6–31+G** level[9, 10]. Due to the strong interaction between water and lithium cation, solvation effect is accounted for using a hybrid approach[11, 12], where the first solvation shell (2 ~ 4 water molecules, depending on the chemical environment) is described explicitly[13], and the remainder treated using a standard polarized continuum model (PCM)[14]. We note that similar choices of computational prescription have been suggested recently in literature when studying the solvation structure of lithium salts[15].

For each chemical species, the total free energy is computed as follows

$$G(\epsilon) = E(\epsilon) + G_{\mathrm{sol}}(\epsilon) + G_{\mathrm{vib}}(\epsilon) \quad (1)$$

where ϵ is the relative permittivity, E is the total energy of the molecule (including any explicit solvent molecules), G_{sol} is the solvation free energy computed by PCM, and G_{vib} is the vibrational free energy obtained from the vibrational analysis. The free energy change of each reaction is then the difference between the total free energy of the products and the reactants. In order to simulate the effect of lithium salt at different concentrations, $\epsilon = 80, 20, \text{and } 10$ are used for the calculation. $\epsilon = 80 \text{ and } 20$ represents $c(\mathrm{Li}^+) \sim 1\mathrm{m}$ and $20\mathrm{m}$, respectively. These choices of ϵ have been experimentally measured on similar lithium salts[16].

Supplementary Note 2. Energetics analysis

We first estimate the thermodynamic energy consumption of our system using the onset potential of PAQ reduction and PAQ–CO_2 adduct oxidation, with theoretical capacity and 100% efficiency (Supplementary Fig. 21).

Cathodic onset potential of PAQ (V_1) = –0.618 V vs Ag/AgCl

Anodic onset potential of PAQ–CO_2 (V_2) = –0.262 V vs Ag/AgCl

Theoretical capacity of PAQ (C) = 2 mol CO_2 per mol PAQ

Number of charges transferred (n) = 2 mol per mol PAQ

Faradic constant (F) = 96485 C mol^{-1}

Energy consumption = $(V_2–V_1)$ nF/ C = 34.35 kJ per mol CO_2

We then calculate the energy consumption using the experimental data carried out using a **constant current capture and a constant current–constant voltage release under simulated flue gas** (shown in Supplementary Fig. 20b). Each capture/release step has a time scale of ~15 min. If $LiFePO_4$ is used as the counter electrode, the system will consume energy when capturing CO_2 and output energy when releasing CO_2. We assume the energy during release can be recovered. The results are presented as Supplementary Fig. 22.

Energy consumed during CO_2 capture = 742 J g^{-1} PAQ

Energy released during CO_2 release = 357 J g^{-1} PAQ

Amount of CO_2 captured = 6.846 mmol g^{-1} PAQ (93% Coulombic efficiency)

Net energy consumption = 385 J g^{-1} PAQ = **56 kJ per mol CO_2**

To compare, the advanced monoethanolamine scrubbing process requires ~45 kJ mol^{-1} of electrical equivalent work for regeneration (optimized system)[17]. And solid sorbent materials explored for carbon capture have enthalpies of adsorption of the same order (e.g. metal–organic frameworks have values of ~30–100 kJ mol^{-1}; zeolites 13X is ~50 kJ mol^{-1}. Note that these values are only enthalpies of adsorption, much greater energetic penalties are needed at the process level.)[18].

Supplementary References

1. Wang, G. & Feng, C. Electrochemical polymerization of hydroquinone on graphite felt as a pseudocapacitive material for application in a microbial fuel cell. *Polymers* **9**, 220 (2017).
2. Shao, Y. et al. Advances in molecular quantum chemistry contained in the Q–Chem 4 program package. *Mol. Phys.* **113**, 184–215 (2015).
3. Riplinger, C. & Neese, F. An efficient and near linear scaling pair natural orbital based local coupled cluster method. *J. Chem. Phys.* **138**, 034106 (2013).
4. Neese, F. The ORCA program system. *Wiley Interdiscip. Rev. Comput. Mol. Sci.* **2**, 73–78 (2012).
5. Becke, A. D. Density–functional thermochemistry. III. The role of exact exchange. *J. Chem. Phys.* **98**, 5648–5652 (1993).
6. Hariharan, P. C. & Pople, J. A. The influence of polarization functions on molecular orbital hydrogenation energies. *Theor. Chim. Acta* **28**, 213–222 (1973).
7. Hehre, W. J., Ditchfield, R. & Pople, J. A. Self–consistent molecular orbital methods. XII. Further extensions of Gaussian–type basis sets for use in molecular orbital studies of organic molecules. *J. Chem. Phys.* **56**, 2257–2261 (1972).
8. Clark, T., Chandrasekhar, J., Spitznagel, G. W. & Schleyer, P. V. R. Efficient diffuse function-augmented basis sets for anion calculations. III. The 3–21+G basis set for first–row elements, Li–F. *J. Comput. Chem.* **4**, 294–301 (1983).
9. Perdew, J. P., Burke, K. & Ernzerhof, M. Generalized gradient approximation made simple. *Phys. Rev. Lett.* **77**, 3865 (1996).
10. Grimme, S. Semiempirical GGA–type density functional constructed with a long–range dispersion correction. *J. Comput. Chem.* **27**, 1787–1799 (2006).
11. Lee, M. S., Salsbury Jr, F. R. & Olson, M. A. An efficient hybrid explicit/implicit solvent method for biomolecular simulations. *J. Comput. Chem.* **25**, 1967–1978 (2004).
12. Lee, M. S. & Olson, M. A. Evaluation of Poisson solvation models using a hybrid explicit/implicit solvent method. *J. Phys. Chem. B* **109**, 5223–5236 (2005).
13. Takeuchi, M. et al. Free–energy and structural analysis of ion solvation and contact ion–pair formation of Li^+ with BF_4^- and PF_6^- in water and carbonate solvents. *J. Phys. Chem. B* **116**, 6476–6487 (2012).
14. Truong, T. N. & Stefanovich, E. V. A new method for incorporating solvent effect into the classical, ab initio molecular orbital and density functional theory frameworks for arbitrary shape cavity. *Chem. Phys. Lett.* **240**, 253–260 (1995).
15. Chapman, N., Borodin, O., Yoon, T., Nguyen, C. C. & Lucht, B. L. Spectroscopic and density functional theory characterization of common lithium salt solvates in carbonate electrolytes for lithium batteries. *J. Phys. Chem. C* **121**, 2135–2148 (2017).
16. Gavish, N. & Promislow, K. Dependence of the dielectric constant of electrolyte solutions on ionic concentration: A microfield approach. *Phys. Rev. E* **94**, 012611 (2016).
17. Wang, M., Hariharan, S., Shaw, R. A. & Hatton, T. A. Energetics of electrochemically mediated amine regeneration process for flue gas CO_2 capture. *Int. J. Greenh. Gas. Con.* **82**, 48–58 (2019).
18. Hu, Z., Wang, Y., Shah, B. B. & Zhao, D. CO_2 capture in metal–organic framework adsorbents: an engineering perspective. *Adv. Sustain. Syst.* **3**, 1800080 (2019).
